# Harnessing the murine inner cell mass mechanical environment enhances derivation of *in vitro* nascent primitive endoderm precursor cells

**DOI:** 10.1242/dev.205226

**Published:** 2026-06-11

**Authors:** Elena Corujo-Simon, Siiri I. Salomaa, Lawrence E. Bates, Ayaka Yanagida, William Mansfield, Hannes Becher, Jennifer Nichols, Kevin J. Chalut

**Affiliations:** ^1^ Wellcome – MRC Stem Cell Institute, University of Cambridge, Jeffrey Cheah Biomedical Centre, Puddicombe Way, Cambridge CB2 0AW, UK; ^2^ Institute of Genetics and Cancer, University of Edinburgh, Western General Hospital, Crewe Road South, Edinburgh EH4 2XU, UK; ^3^ Department of Veterinary Anatomy, The University of Tokyo, Bunkyo-ku, Tokyo 113-8657, Japan; ^4^ Department of Physiology, Development and Neuroscience, University of Cambridge, Tennis Court Road, Cambridge CB2 3EG, UK; ^5^ The Loke Centre for Trophoblast Research, Physiology Building, Downing Street, Cambridge CB2 3EG, UK

**Keywords:** Primitive endoderm, Mechanical properties, Hydrogels, FGF signalling, Integrins, Fibronectin, Extracellular matrix, Mouse

## Abstract

Stem cell-based models resembling murine blastocysts represent a useful system to investigate subsequent developmental processes. While existing cell lines derived from epiblast and trophectoderm can be aggregated to form ‘blastoids’, some previously tested *in vitro* cultured extra-embryonic endoderm cells tended to progress to later stages of development, so integrated inefficiently into blastoids. We attempted to capture the precursor population for extra-embryonic endoderm *in vitro* by reproducing the mechanical environment of the *in vivo* peri-implantation embryo as closely as possible. We investigated expression of candidate cell adhesion receptor integrins in the blastocyst inner cell mass, and from this information we assembled an extracellular matrix intended to support primitive endoderm growth by promoting signalling pathways responsible for specification of this lineage. In addition, inner cell mass cells from blastocysts were plated on soft or stiff substrates to investigate whether an appropriate mechano-environment could enhance their self-renewal as primitive endoderm in culture. We could expand nascent primitive endoderm cell lines over several passages, which provided a reproducible, albeit short-term, system sufficient to identify some essential requirements for early primitive endoderm expansion and function.

## Teaser

**Summary:** Manipulation of substrate stiffness and integrin expression in mouse cell cultures reveals *in vitro* conditions that can transiently sustain a cell identity equivalent to that of *in vivo* early primitive endoderm precursors.

## INTRODUCTION

Mouse pre-implantation development is characterised by the appearance of three lineages: trophectoderm (TE), the precursor of the placenta; epiblast (EPI), which will form the embryo proper; and primitive endoderm (PRE), giving rise to the yolk sac and gut endoderm as well as being required for patterning the EPI (Schrode et al., 2013). Acquisition of these three lineages is accomplished by two sequential cell fate decisions during blastocyst development. The first takes place at embryonic day (E) 2.5, the morula stage, when outer cells polarise and differentiate into TE while apolar cells located on the inside become the inner cell mass (ICM) (Copp, 1978; Nishioka et al., 2008; Strumpf et al., 2005). The second lineage segregation occurs within the ICM, initiating at around E3.5. At this stage, ICM cells are defined as ‘co-expressing’ EPI markers, such as OCT4 (POU5F1) and NANOG (Dietrich and Hiiragi, 2007), and the PRE markers GATA6 and PDGFRA, at heterogeneous levels (Chazaud et al., 2006; Plusa et al., 2008). EPI precursors specify first, gradually losing expression of PDGFRA and GATA6 (Grabarek et al., 2012). They secrete FGF4 into the ICM microenvironment, promoting PRE specification in neighbouring ICM cells at around E4.0 (Kang et al., 2017; Molotkov et al., 2017); these cells are no longer positive for NANOG, but still express OCT4, GATA6 and PDGFRA (Mohammed et al., 2017; Nowotschin et al., 2019). PRE precursors remain plastic and able to dedifferentiate into EPI cells as long as they maintain OCT4 expression, while simultaneously initiating the expression of the later PRE markers SOX17, GATA4 and SOX7 (Artus et al., 2011; Grabarek et al., 2012; Linneberg-Agerholm et al., 2024). The EPI/PRE cell fate decision is complete at E4.5. EPI cells lose NANOG expression as they transition from naïve to primed pluripotency (Chambers et al., 2003), and PRE cells downregulate OCT4 and express SOX7 (Artus et al., 2011).

At this stage, EPI and PRE have segregated physically in the ICM, and PRE cells overlying the EPI begin to migrate along the inside of the mural TE to become parietal endoderm (Grabarek et al., 2012). Descendants of the PRE that remain in contact with EPI become visceral endoderm (VE). Following implantation, VE cells secrete signals that induce anterior-posterior patterning of the EPI to begin gastrulation (Kimura et al., 2000; Perea-Gomez et al., 2001; Srinivas et al., 2004; Thomas et al., 1998). Both EPI/PRE differentiation and segregation occur within the ICM physical microenvironment, characterised by the presence of the extracellular matrix (ECM), a network formed by laminin and fibronectin (FN), among other components. The mechanical interaction between cells and ECM through integrin-mediated cell adhesion is increasingly being characterised and considered to play a role at this stage of development (Kim et al., 2022); differences in mechanical cell surface fluctuations regulate EPI/PRE sorting, while disruption of integrins, responsible for the connection between cells and the ECM, prevents segregation of PRE as a monolayer at E4.5 (Kim et al., 2022; Yanagida et al., 2022).

Stem cell-based embryo models representing early embryonic cell lineages have been developed to enable experiments *in vitro.* Mouse embryonic stem cells (mESCs) recapitulating the *in vivo* EPI were the first lines to be derived and characterised (Boroviak et al., 2014; Evans and Kaufman, 1981; Martin, 1981). However, cell lines aiming to model the blastocyst extra-embryonic lineages, including trophoblast stem cells from TE (Tanaka et al., 1998) and extra-embryonic endoderm (XEN) cells derived from PRE (Kunath et al., 2005) were frequently more representative of post-implantation lineages (Garg et al., 2016). XEN cells mostly recapitulate the parietal endoderm lineage, with limited contribution to VE in chimeras. They are thus unsuitable for generation of organoids representing pre-implantation mammalian development, known as ‘blastoids’ (Rivron et al., 2018).

To attempt to overcome inadequacies of PRE contribution to blastoids, mESCs were cultured under extended pluripotent conditions (Sozen et al., 2019) or by inducing expression of PRE markers prior to aggregate formation (Amadei et al., 2021). However, cell lines with either of these modifications could not be individually characterised or maintained over passages. Recently, primitive XEN cells (Zhong et al., 2018) and PRE stem cells (Ohinata et al., 2022) derived from mouse embryos, or naïve endoderm cells from direct differentiation of ESCs (Anderson et al., 2017) or from early embryos or isolated ICMs subjected to stringently established lineage induction regimes (Linneberg-Agerholm et al., 2024) have been derived. Previously, some protocols had focused on improving media conditions or overexpressing GATA transcription factors to differentiate mESCs into PRE cells (Fujikura et al., 2002; Niakan et al., 2013; Ohinata et al., 2022; Schroter et al., 2015; Shimosato et al., 2007; Vrij et al., 2022; Zhong et al., 2018).

With the aim of optimising the mechano-environment and recapitulating the *in vivo* condition as closely as possible, we characterised integrin expression within the ICM to choose the most suitable ECM combination. We also modified substrate stiffness to mimic the cell-extrinsic cues necessary to support naïve primitive endoderm (NPRE) propagation. We generated cell lines from mouse embryos that could sustain early NPRE identity for up to four passages, equivalent to blastocyst PRE precursors. Even though long-term self-renewal could not be achieved in our conditions, characterisation of ICM mechano-environment enhances knowledge of the interaction between signalling pathways and the substrate, improving basic understanding of PRE cell specification.

## RESULTS

### Integrin α5β1 activity is necessary for PRE cell fate specification

**Fig. 1.**
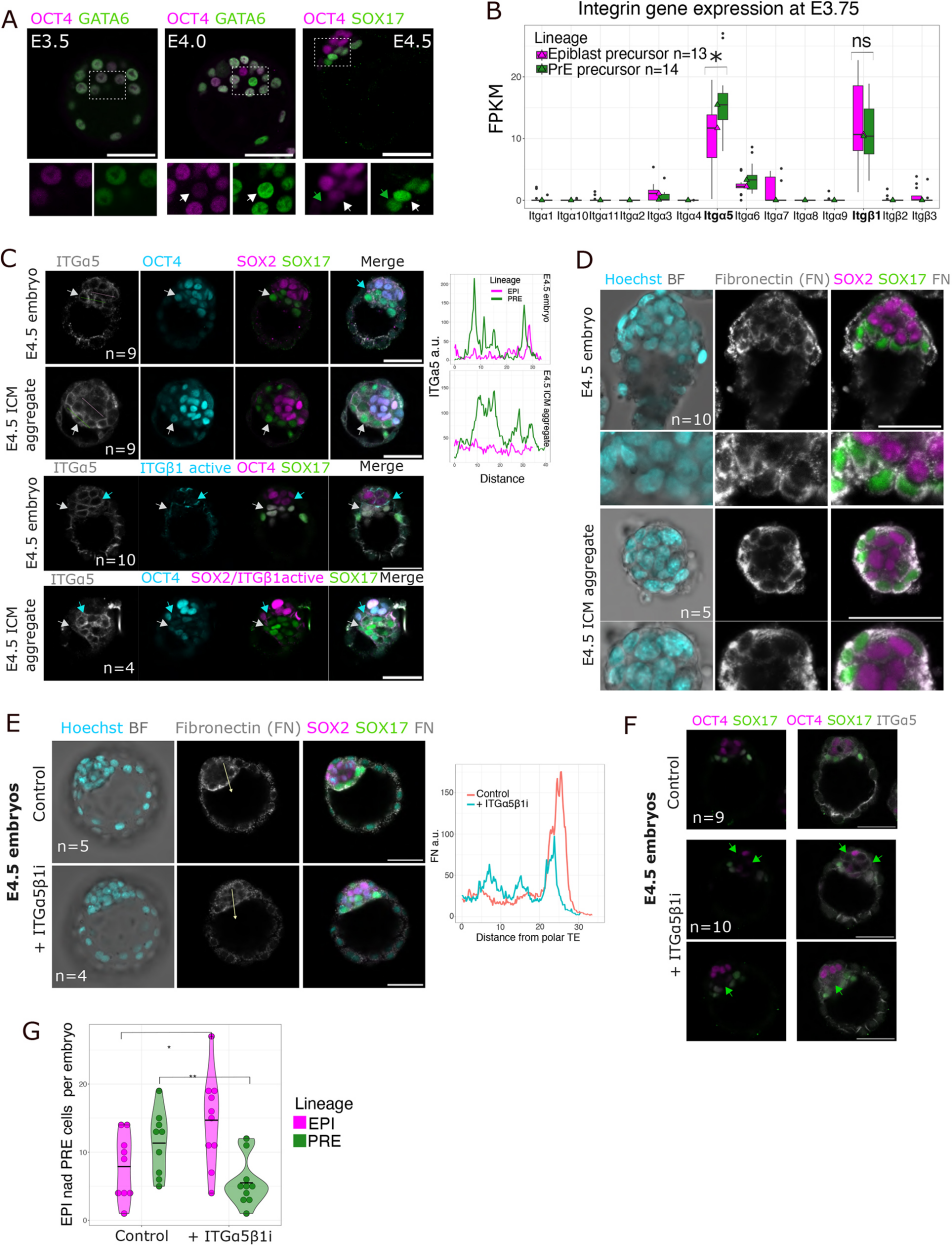
**Differential integrin expression and activity between EPI and PRE during blastocyst development.** (A) Representative confocal images of pre-implantation mouse embryos from E3.5 to E4.5 immunostained for OCT4 (magenta; EPI) and GATA6/SOX17 (green; PRE). Boxed areas are shown at higher magnification beneath. White arrows indicate GATA6^+^ cells retaining residual expression of OCT4, green arrows indicate GATA6^+^, OCT4^−^ cells. (B) Integrin gene expression from single ICM cells (EPI and PRE precursors) at E3.75 from three embryos. Statistical significance was assessed using a two-tailed Student's *t*-test (*P<0.05). ns, not significant (*P*>0.05). Box limits represent 5 FPKN, horizontal line shows median of the distribution and whiskers outline the horizontal limits of data points. (C) Left: Representative confocal images of E4.5 mouse embryos or E4.5 ICM aggregates – made of three E3.5 ICMs – immunostained for ITGα5 (white), active ITGβ1 (cyan or magenta), OCT4 (cyan or magenta), SOX2 (magenta) and SOX17 (green). White arrows indicate higher ITGα5 expression, cyan arrows indicate localised active ITGβ1 expression. Right: Representative intensity profiles of ITGα5 across the EPI (marked as a magenta line on the confocal image on the left) and across the PRE (marked as a green line on the confocal image on the left). a.u., arbitrary units. (D) Representative confocal images of E4.5 mouse embryos and ICM aggregates immunostained for fibronectin (FN) (grey), SOX2 (magenta) and SOX17 (green). Nuclei are labelled with Hoechst. (E) Left: Representative confocal images of E4.5 mouse embryos cultured in the absence or presence of antibodies to block ITGα5β1 activity (CD29 and CD49e) and immunostained for SOX2 (magenta), SOX17 (green) and FN (grey). Right: Representative intensity profile of FN in control (cyan) and treated (red) embryos from the polar TE across the EPI into the PRE and the blastocoele (marked as lines on the confocal images on the left; arrowhead indicates the end of measurement). (F) Representative confocal images of E4.5 mouse embryos cultured in the absence or presence of antibodies to block ITGα5β1 activity (CD29 and CD49e) and immunostained for OCT4 (magenta), SOX17 (green) and ITGα5 (grey). Green arrows point towards central ICM cells expressing neither OCT4 nor SOX17. (G) Violin plot showing the average total cell number of each lineage in control and ITGα5β1 inhibitor-treated (ITGα5β1i) embryos. The numbers of embryos used for each experiment are written within the relevant panels. Statistical significance was assessed using a two-tailed Student's *t*-test (*P<0.05, **P<0.005). Scale bars: 50 µm.

Our primary objective was to manufacture a system to support self-renewal of an *ex vivo* PRE population by investigating the combination of ECM and mechanical microenvironment most permissive for PRE culture. We therefore characterised ICM integrin expression to choose the most suitable ECM, and modified substrate stiffness to mimic the *in vivo* condition. We studied expression of integrins in the mouse ICM at E4.0, just before ICM cells have assumed exclusive expression of EPI versus PRE markers or completed physical segregation (Fig. 1A, Fig. S1A). Importantly, integrins form heterodimers of an alpha and a beta subunit, which have high specificity for different ECM components (Chastney et al., 2021; Hynes, 2002). Hence, the nature of integrin subunit expression can inform the optimal choice of ECM required to maintain PRE identity. We discovered that *Itga5* and *Itgb1* were expressed at a significantly higher level than other possible subunits at E4.0, with *Itga5* expression enhanced in PRE precursors compared with EPI progenitors at this stage (Fig. 1B). To confirm these differences, we examined protein expression for ITGα5 and ITGβ1 as well as activity of ITGβ1 at E3.5, E4.0 and E4.5. At E4.5, the difference in intensity of ITGα5 became more evident, and active ITGβ1 was clearly localised to the boundary between EPI and PRE (Fig. 1C, Fig. S1B). Notably, the integrin heterodimer α5β1 preferentially binds to the ECM protein FN (Chastney et al., 2021; Pacifici et al., 1992). Given this potential ECM connection in the establishment of the blastocyst, we next investigated the role of FN.

From E3.5 to E4.0, FN was found ubiquitously expressed in the ICM with no clear difference between EPI and PRE precursors (Fig. S1C). However, as with the expression pattern of ITGα5, FN expression was clearly localised in the sorted PRE beside the blastocoel in E4.5 embryos and outer cells of E4.5 ICM aggregates (Fig. 1D, Fig. S1D). Since our interest is to find the ECM that will best support PRE cell growth *ex vivo*, we explored further the interdependency between integrins and ECM. E3.5 mouse blastocysts were treated with a combination of function-blocking antibodies against ITGα5β1 and changes in the expression of FN were analysed. Clear localisation of FN in PRE cells at E4.5 was lost when combined with ITGα5β1 inhibition, remaining ubiquitously expressed in the ICM as observed in earlier stages of development (Fig. 1E, Fig. S1C). Similarly, ITGα5 was concentrated in the PRE in control embryos, but expressed equally throughout the ICM when ITGα5β1 inhibition was applied, coincident with destabilised marker expression for EPI and PRE (Fig. 1F). Quantification analysis revealed that ITGα5β1 inhibition reduced cell numbers in the PRE lineage (Fig. 1G, Fig. S1E), whereas total cell numbers in the ICM did not differ significantly (Fig. S1F). The decrease in the number of PRE cells was confirmed through additional biological repeats and using ATN-161, a small peptide able to inhibit the activity of the integrin α5β1 heterodimer (Khalili et al., 2006) (Fig. S1G-I). These results suggest that functional integrin activity is necessary for efficient PRE lineage acquisition.

### Integrin activity affects lineage acquisition via FGF/ERK signalling

**Fig. 2.**
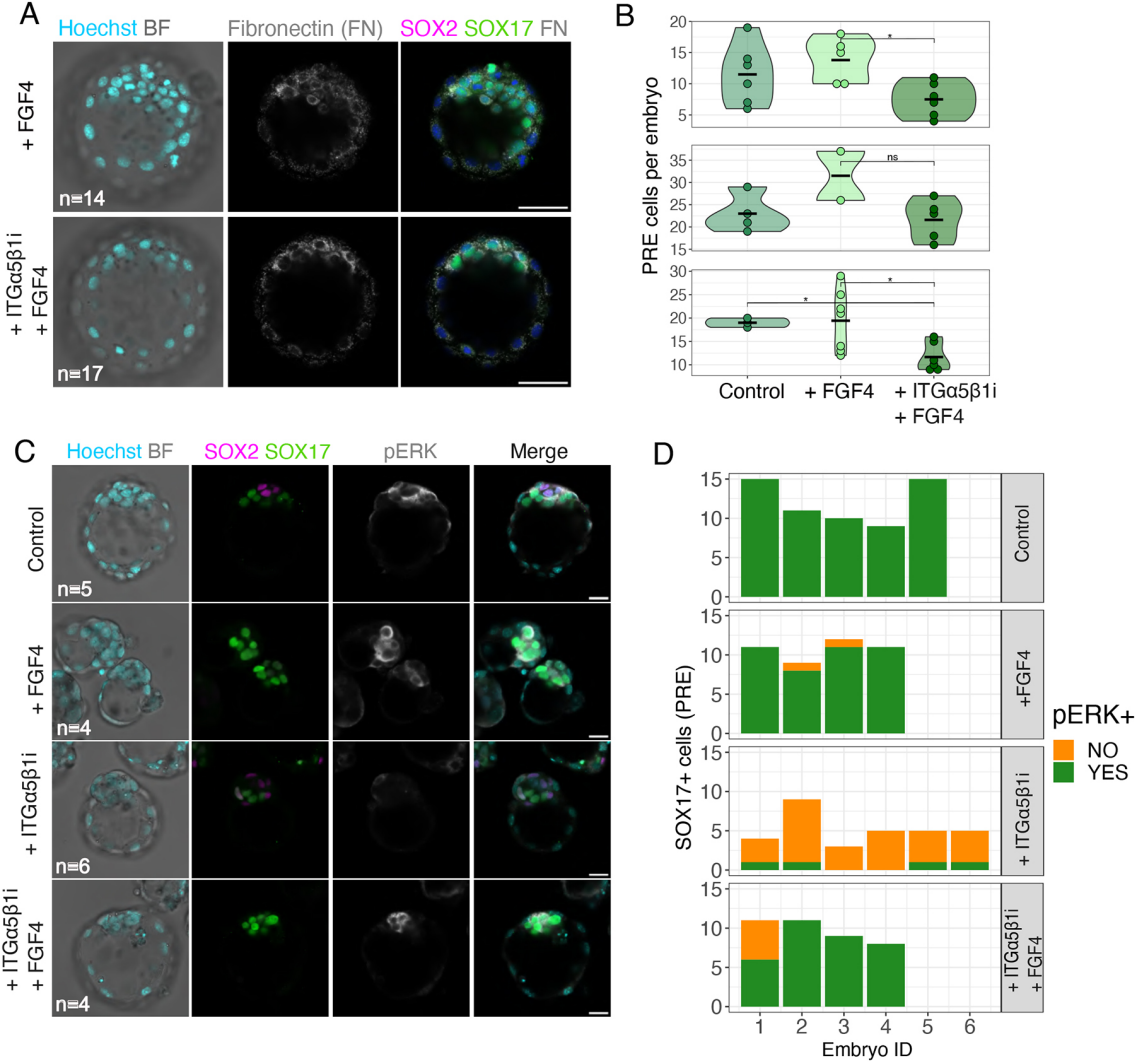
**Integrin activity affects PRE lineage acquisition through FGF/ERK signalling.** (A) Representative confocal images of E4.5 mouse embryos cultured with FGF4 in the absence or presence of integrin inhibiting antibodies. Embryos were immunostained for FN (grey), SOX2 (magenta) and SOX17 (green); nuclei are labelled with Hoechst. (B) Violin plots of three experiments comparing the average number of PRE cells per embryo in the untreated control condition, FGF4 treated, and under FGF4 and integrin inhibiting treatment. Statistical significance was assessed using a two-tailed Student's *t*-test (*P<0.05). ns, not significant (*P*>0.05). (C) Representative confocal images of control E4.5 mouse embryos, FGF4-treated embryos, embryos treated with integrin activity inhibiting antibodies, and embryos cultured under the combination of both FGF4 and integrin inhibition. Embryos were immunostained for SOX2 (magenta), SOX17 (green) and pERK (white). (D) Stacked bar plots quantifying the number of pERK^+^ (green) and pERK^−^ (orange) ICM cells per embryo in the different conditions presented in C. Scale bars: 50 µm.

Based on the effect of integrin α5β1 inhibition in diminishing the number of PRE cells, we next explored a potential interaction between integrin activity and FGF/ERK, which is an essential signal for PRE specification in the mouse embryo (Fafilek et al., 2018). We treated E3.25 embryos with 1 μg/ml FGF4 and integrin function-blocking antibodies and compared their ICM composition to embryos cultured with FGF4 alone (Fig. S2). All ICM cells become PRE in both instances; however, we observed a significant decrease in the number of PRE cells in embryos subjected to integrin inhibition (Fig. 2A,B).

It was recently shown that pERK is required for PRE specification in the mouse blastocyst (Azami et al., 2019). We observed high levels of pERK in PRE cells in the control and in all ICM/PRE cells upon treatment with FGF4, independently of integrin inhibition, whereas in embryos treated with integrin function-blocking antibodies only, pERK levels were decreased in the entire ICM (Fig. 2C). Some SOX17-positive PRE cells observed in integrin-inhibited embryos were negative for pERK, suggesting they may have already been specified prior to treatment (Fig. 2D). Our data suggest that localised ITGα5β1 activity is required in the ICM from E3.5 to E4.5 to achieve PRE cell lineage acquisition, thus implicating this essential receptor for FN in regulating FGF/ERK activity and fate specification in PRE cells. While these results demonstrate that integrin signalling is required for ERK activation, other mechanisms, such as integrin-mediated clustering of FGF receptors or regulation of receptor trafficking, cannot be excluded.

Based upon the strength of the pERK response to FGF4 administration even in the presence of ITGα5β1 inhibition (Fig. 2C,D), we conclude that FN, the primary ligand for ITGα5β1, would be the optimal ECM upon which to derive and maintain early PRE cells *ex vivo*.

### Soft FN-coated substrates promote PRE growth

**Fig. 3.**
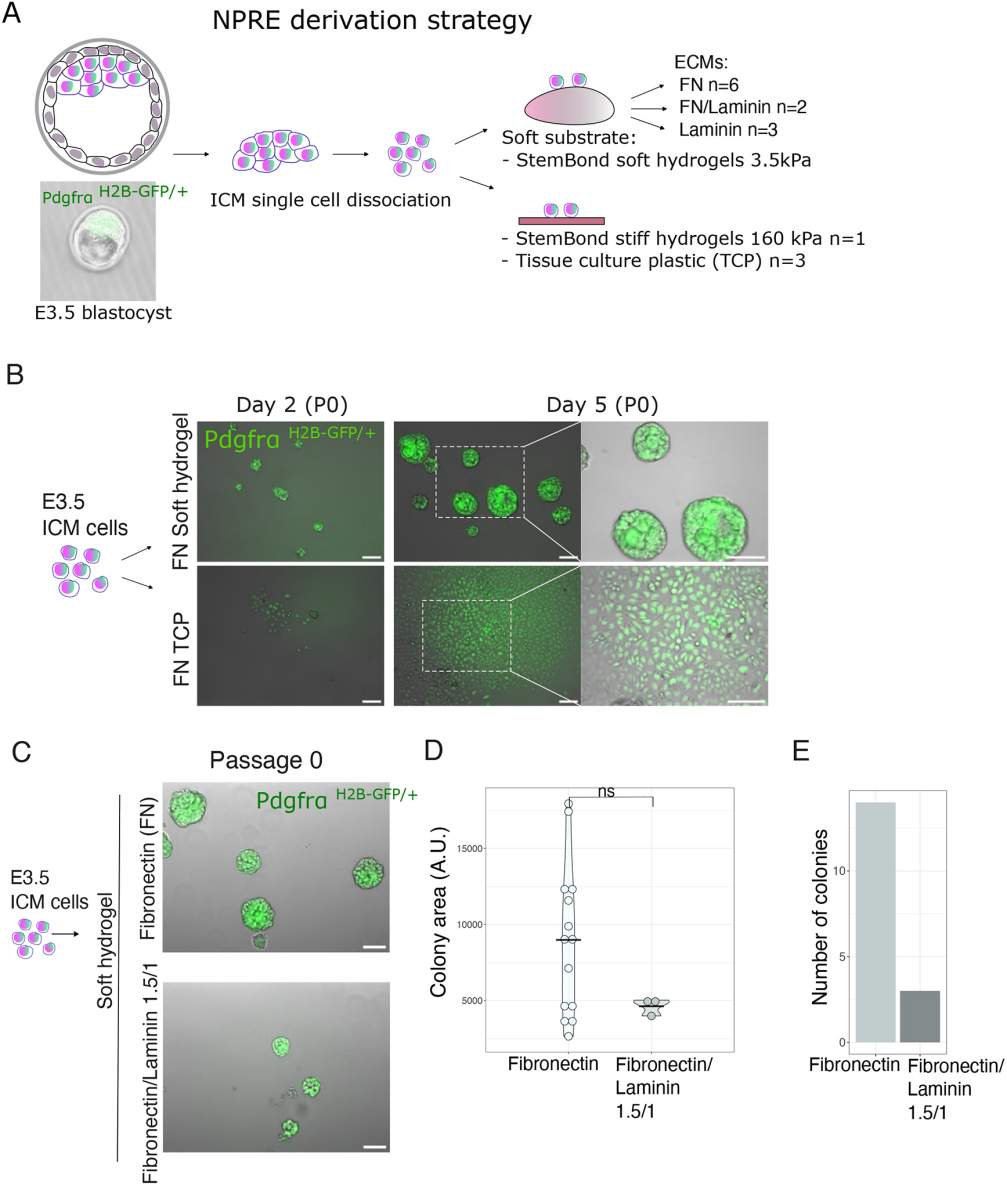
**Soft FN-coated substrates promote PRE growth.** (A) Diagram illustrating the NPRE derivation strategy. Immunosurgically isolated ICMs from Pdgfrα^H2B-GFP/+^ mouse blastocysts are dissociated into single cells and seeded onto substrates with different stiffness and distinct ECM combinations. The number of biological repeats is specified in the right side of the figure. The derivation was performed on soft hydrogels a total of 14 times (nine on FN, two on FN/laminin and three on laminin), on stiff hydrogels twice, and on TCP three times. (B) Pdgfrα^H2B-GFP/+^ colonies (top) or single cells (bottom) are shown, on distinct substrate stiffness at day 2 and day 5 following seeding and culture in NPRE media. (C) Pdgfrα^H2B-GFP/+^ colonies growing on soft hydrogels coated with FN (top) or a combination of FN and laminin (1.5/1; bottom). (D) Violin plot quantifying the colony area of Pdgfrα^H2B-GFP/+^ colonies growing on FN or the combination of FN and laminin (1.5/1) as shown in C. Statistical significance was assessed using a two-tailed Student's *t*-test. ns, not significant (*P*>0.05). A.U., arbitrary units. (E) Stacked bar plots quantifying the number of colonies on FN compared to FN/laminin. Scale bars: 50 µm.

Since previous attempts to propagate PRE cells outside the embryo have been primarily based on tissue culture plastic (TCP), a mechanical environment less flexible than that of the embryo, we adapted the culture medium and substrate stiffness to enable growth of single E3.5 ICM cells into NPRE colonies *ex vivo*. To optimise the mechanical environment, we compared the effects of stiff TCP with stiff or soft StemBond hydrogels (Labouesse et al., 2021) for capture of NPRE in culture. PDGFRα^H2B-GFP/+^ embryos (Hamilton et al., 2003) allowed us to follow PRE appearance by fluorescence while testing the culture media on TCP and on soft (3.5 kPa) and stiff (160 kPa) hydrogels with different ECM combinations to examine potential differences between the substrates under the same media conditions (Fig. 3A).

RPMI (formulated at Rosewell Park Memorial Institute, NY, USA) was used as basal medium and supplemented with fetal bovine serum, sodium pyruvate, β-mercaptoethanol, leukaemia inhibitory factor (LIF), CHIR99021 (Chiron; GSK3 inhibitor), PDGF-AA, Gö6983 (pan-PKC inhibitor), FGF4 and heparin (see Materials and Methods). Removal of LIF killed the cells; withdrawal of Chiron resulted in poor survival (Fig. S3A). On stiff hydrogels, ICMs were unable to attach and produced hollow structures comprising mostly GATA3^+^ cells, assumed to be TE, whereas they formed more-solid, SOX17^+^ structures on soft hydrogels (Fig. S3B). On TCP, ICMs attached and formed outgrowths of the three pre-implantation fates (TE, EPI and PRE), while on soft hydrogels there was no detectable TE (Fig. S3C). Under FGF4 treatment, ICM outgrowths on soft hydrogels produced predominantly PRE cells, while those on TCP also contained a TE population (Fig. S3C). On soft hydrogels coated with FN, single ICM cells form NPRE round colonies, in contrast to NPRE cells on TCP coated with FN, which appeared spread out and motile, resembling XEN cells (Fig. 3B, Fig. S3D). On stiff hydrogels, single cells did not attach at all. Thus, we focused on using soft hydrogels for culturing NPRE cells. To further validate the use of FN on the hydrogels, we compared coating with FN, laminin or a mixture of both. Starting from the same seeding density, more numerous and larger colonies appeared on FN compared to FN/laminin or just laminin (Fig. 3C-E, Fig. S3E). Taken together, our data indicate that FN-coated soft hydrogels support growth of NPRE cells.

### NPRE cells resembling *in vivo* E4.5 PRE can be propagated using FN-coated soft substrates in media containing LIF, PDGF-AA and Chiron

**Fig. 4.**
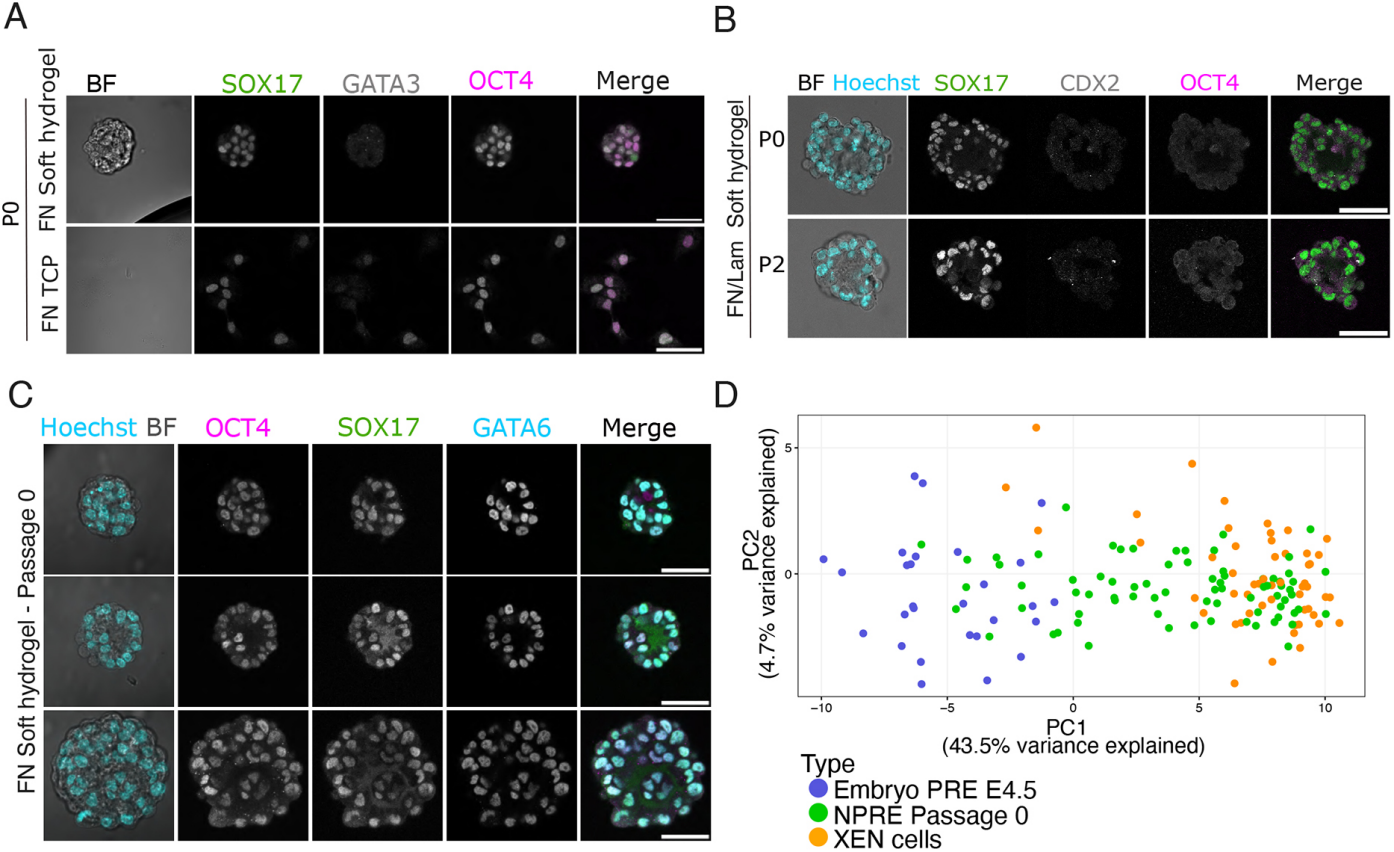
**NPRE resembling *in vivo* E4.5 PRE can be derived using FN-coated soft substrates in controlled media conditions.** (A) Representative confocal images of NPRE colonies at P0 grown on FN-coated soft hydrogels (top) or TCP (bottom) and immunostained for OCT4 (magenta), SOX17 (green) and GATA3 (grey). (B) Representative confocal images of SOX17^+^ cells grown on soft hydrogels coated with a mixture of FN and laminin (1.5/1; FN/Lam) and immunostained for SOX17 (green), OCT4 (magenta) and CDX2 (white) immediately (top) or after two passages (bottom). (C) Representative confocal images of NPRE colonies at P0 grown on FN-coated soft hydrogels and immunostained for OCT4 (magenta), SOX17 (green) and GATA6 (cyan). A minimum of five colonies were imaged per passage per ECM. (D) Principal component analysis comparing gene expression of E4.5 PRE cells, P0 NPRE cells and XEN cells. Scale bars: 50 µm.

To further validate our approach for the culture of NPRE cells, we performed molecular characterisation. On soft hydrogels, NPRE cells expressed the ICM marker OCT4 and the PRE marker SOX17; TE markers were supressed; NPRE cells on TCP coated with FN also expressed GATA3 (Fig. 4A). Furthermore, NPRE colonies on FN/laminin did not express OCT4 (Fig. 4B), which is expressed in PRE cells *in vivo* until peri-implantation (Artus et al., 2011; Grabarek et al., 2012), thus indicating a more advanced developmental state. Further characterisation of passage 0 (P0) NPRE colonies grown in our preferred media (see Materials and Methods; Table S3) and substrate conditions confirmed expression of OCT4 and the PRE markers SOX17 and GATA6 (Fig. 4C). Gene expression analysis by single-cell RNA sequencing (scRNA-seq) showed that P0 NPRE cells represent a heterogeneous population comprising embryonic E4.5 PRE cells and XEN-like cells, similar to other published cell lines (Ohinata et al., 2022) (Fig. 4D, Fig. S4A). These results confirmed that PDGFRA^+^ cells grown on FN-coated soft hydrogels from single E3.5 ICM cells resemble the *in vivo* PRE at E4.0-E4.5 (Fig. S4B). Our comparative analysis strongly suggests that FN-coated soft hydrogels combined with our media formulation show promising support for NPRE cells.

### NPRE maintenance over passages is dependent on the concentration of FGF4

**Fig. 5.**
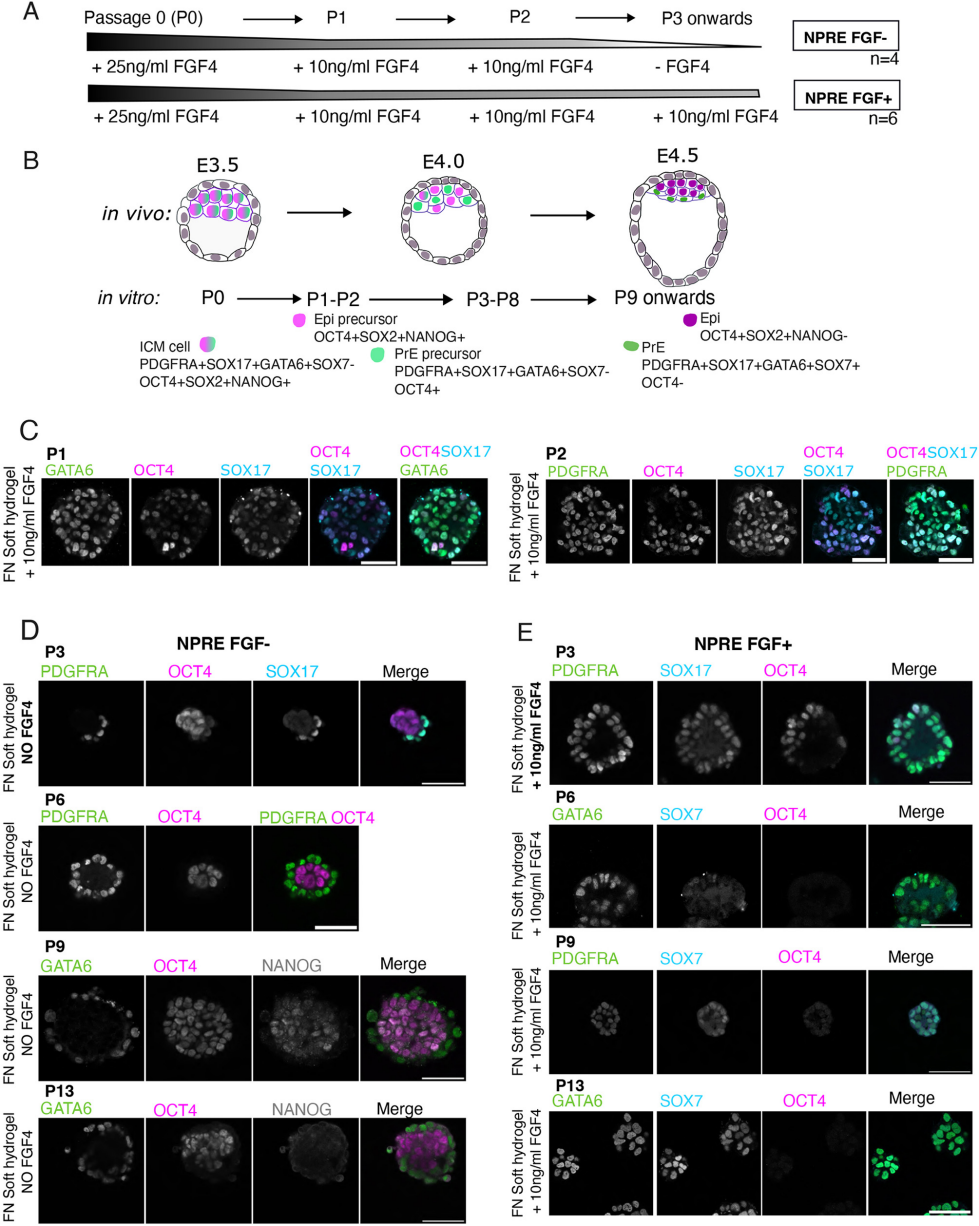
**NPRE cells develop over passages similarly to the ICM in the pre-implantation mouse embryo.** (A) Schematic illustrating the NPRE passage strategy with varying concentrations of FGF4. A minimum of five colonies were imaged per passage per condition. (B) Schematic of mouse embryo blastocyst stages and their equivalent NPRE cell passage according to expression of EPI and PRE markers. (C) Representative confocal images of NPRE colonies during the early passages 1 and 2 (P1, P2), immunostained for the PRE markers GATA6 (green), SOX17 (cyan) and PDGFRA (green) as well as OCT4 (magenta). (D,E) Representative confocal images of NPRE cells from P3 onwards in the absence (NPRE FGF−) (D) or presence (NPRE FGF+) (E) of FGF and immunostained for the PRE markers PDGFRA (green), GATA6 (green), SOX17 (cyan), SOX7 (cyan), the pan-ICM marker OCT4 (magenta) and the EPI marker NANOG (white). Scale bars: 50 µm.

Maintenance of the NPRE phenotype during propagation is important to create a cell line that can be used to study the *in vivo* PRE lineage *in vitro* and to make aggregates with the aim of modelling pre-implantation development. To impede NPRE cells from further development and adopting post-implantation fates, we decreased the concentration of FGF4 following the first passage (P1) from 25 ng/ml to 10 ng/ml, then at P3 either removed FGF4 (NPRE FGF−) or maintained it at the lower concentration of 10 ng/ml (NPRE FGF+), suitable for cells in monolayer culture (Fig. 5A, Fig. S5A-C) compared with whole embryos (Fig. 2).

Protein characterisation revealed that NPRE cells from P0 to P2 mimicked the heterogeneous nature of the unspecified E3.5/E4.0 ICM cells, expressing both the pan-ICM marker OCT4 and various PRE markers (Fig. 5B,C). Upon removal of FGF4 at P3, NPRE cells specified to become either PRE-like or EPI-like, as observed by mutually exclusive marker expression (Fig. 5B,D). PRE-like and EPI-like cells co-existed over multiple passages. However, both cell types progressed in development as the passage number increased, as previously reported for EPI-like cells, losing NANOG expression at P13 (Fig. 5D), similarly to the *in vivo* EPI at E4.5 (Chambers et al., 2007). FGF4 removal produced the same effect on NPRE cells grown on TCP substrates: EPI-like cells appeared independently of the substrate upon which they were grown (Fig. S5D,E). In parallel, we derived NPRE cells maintaining FGF4 in the media (Fig. 5E, Fig. S5C). No EPI-like cells were observed in the presence of FGF4. Instead, OCT4 expression was progressively lost following P3 (Fig. 5E), and NPRE FGF+ cells became a homogeneous cell line expressing only PRE markers (Fig. 5E). Similarly to cells grown without FGF, NPRE FGF+ cells developed as they would *in vivo*. By P6, they were expressing SOX7, a late PRE marker detectable *in vivo* from E4.5 (Fig. 5B,E). If successful at reproducing the ICM microenvironment, NPRE cells would be expected to recreate the integrin expression pattern observed *in vivo*. NPRE FGF+ cells displayed high levels of ITGα5 and active ITGβ1 (Fig. S5F). NPRE FGF− cells on hydrogels also reproduced the pattern observed in mouse embryos: EPI-like cells showed no expression of ITGα5β1 while PRE-like cells expressed high levels (Fig. S5F).

In summary, we have devised conditions to capture NPRE cells that resemble the unspecified ICM at E3.5/E4.0 for at least three passages by growing single embryonic ICM cells on FN-coated hydrogels. By removing FGF from these cells, we obtained an *in vitro* model of ICM differentiation into EPI-like and PRE-like cells, whereas maintaining FGF in the media allowed us to produce a model of PRE-like cell specification (Fig. 5B).

### NPRE FGF+ cells can contribute to host blastocyst PRE and aggregate with mESCs

**Fig. 6.**
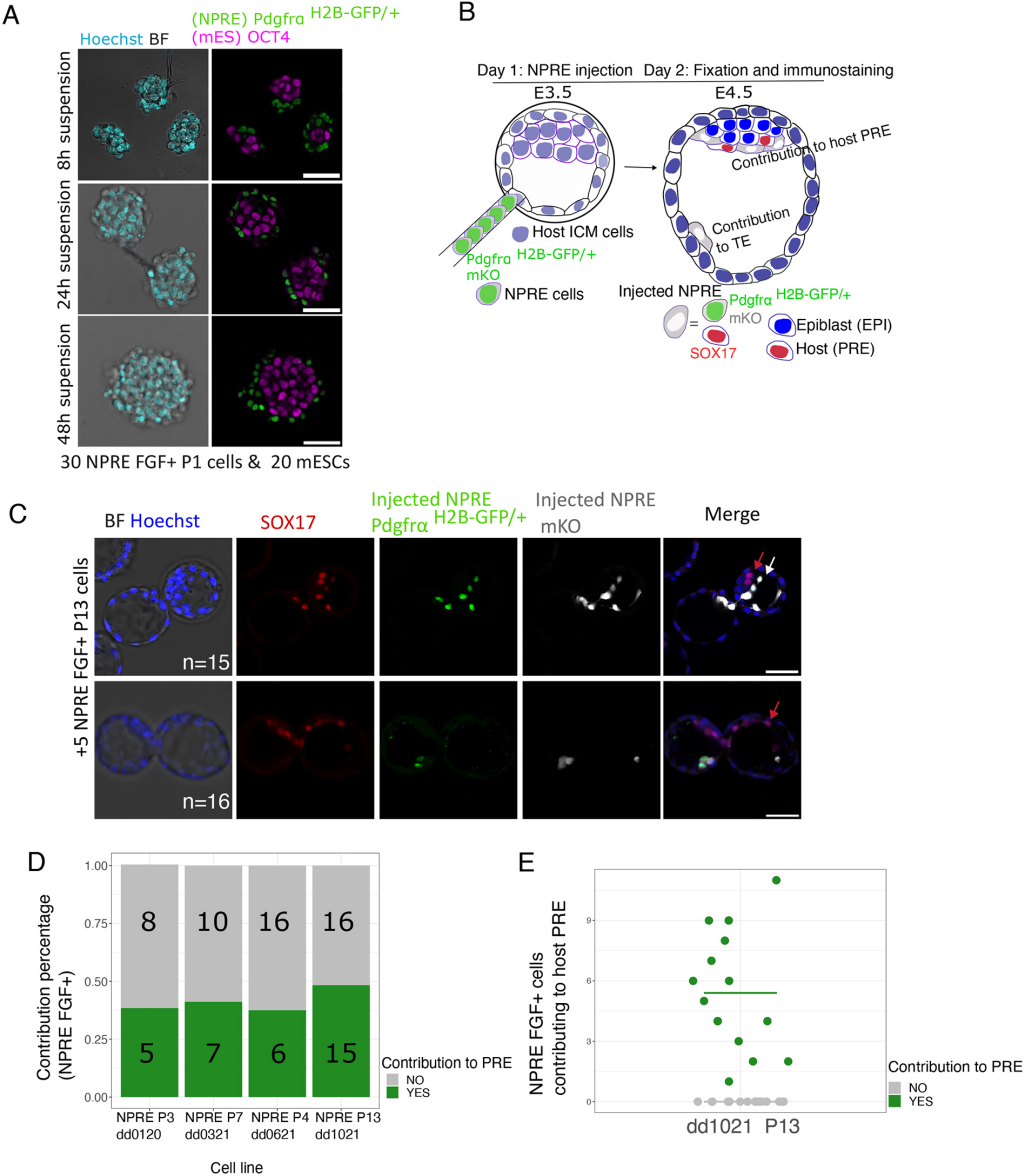
**NPRE FGF+ cells aggregate with mESCs and contribute to host PRE in mouse blastocysts.** (A) Representative confocal images of ICM aggregates made of 30 NPRE cells and 20 mESCs 8, 24 and 48 h after initial seeding. Pdgfrα ^H2B-GFP/+^ FGF+ NPRE cells (green) and mESCs immunostained for OCT4 (magenta) are shown with nuclei stained with Hoechst. (B) Chimera injection diagram. Five NPRE cells were injected into host blastocysts at E3.5. Embryos were allowed to develop until E4.5 prior to fixation and immunostaining. (C) Representative confocal images of injected NPRE cells contributing to the host PRE in the mouse blastocyst (top) or attaching and contributing to the TE (bottom). (D) Quantification of the contribution percentage (percentage of NPRE FGF+ cells contributing to the PRE lineage) across various injections datasets from different passages with the number of injected embryos. (E) Scatter plot quantifying the number of NPRE cells contributing to the host PRE per embryo (green) or to the TE (grey). dd number indicates dataset date for each set of injections. Scale bars: 50 µm.

NPRE cells would be expected to form aggregates with mESCs to reproduce the mouse ICM *in vitro*. We therefore tested their ICM aggregation ability by mixing NPRE FGF+ cells with mESCs and culturing the aggregate in suspension. As early as 8 h after seeding, both cell types formed mixed aggregates (Fig. 6A). Following 24 h culture, NPRE FGF+ cells had segregated to surround the mESCs, recapitulating PRE/EPI sorting at E4.5 *in vivo* (Fig. 6A) (Plusa et al., 2008; Yanagida et al., 2022). The sorted pattern was maintained at 48 h as the aggregate increased in size following cell proliferation of both populations (Fig. 6A). In addition, ICM-like aggregates formed of NPRE FGF+ cells and mESCs also recapitulated the *in vivo* integrin expression at E4.5 (Fig. 1C). ITGα5 expression was localised to the outer layer of NPRE cells and active ITGβ1 signal was higher in the boundary separating the inner mESCs from the outer NPRE (Fig. S6A). To achieve the 60/40 PRE/EPI ratio reported for *in vivo* mouse ICM (Saiz et al., 2016) using NPRE FGF− cells, we separated the cells based on morphology and cultured them as two distinct cell lines for one passage prior to aggregation. Similarly to those made with FGF+ cells, ICM aggregates appeared sorted after 24 h and cells were able to proliferate and form larger aggregates at 48 h (Fig. S6B). The EPI-like component formed rosettes at 48 h, which suggests they represent a later stage of development than the E4.5 *in vivo* EPI (Fig. S6B) (Neagu et al., 2020).

Finally, we investigated the potential of the derived cells to contribute to the PRE of host embryos by injecting five mKO-Pdgfra^H2B-GFP/+^ NPRE FGF+ cells into mouse blastocysts (Fig. 6B, Fig. S6C-E). On the following day, NPRE FGF+ cells had incorporated into the host PRE adjacent to the blastocoel in 48.4% of embryos (Fig. 6C-E). NPRE cells failed to contribute to the ICM if injected at the morula stage (Fig. S6F), implying that the relative immaturity of the host environment was unsupportive of NPRE survival. By contrast, when injected at E3.5 we observed robustness of contribution across NPRE passage number: NPRE FGF+ injections showed contribution to the host PRE, varying from 37.5% to 48.4% using cells from different passages: 13 embryos for P3, 16 for P4, 17 for P7 and 31 for P13 (Fig. 6D, Fig. S6D).

## DISCUSSION

In this study, we have highlighted the importance of the mechanical and biochemical microenvironment of the *in vivo* cell niche to inform *ex vivo* strategies for derivation of cell lines, particularly PRE from the murine ICM. Following sorting at E4.5, EPI and PRE are separated by the basal lamina, an ECM comprising laminin and FN among other proteins, to which they adhere through integrins (Gersdorff et al., 2005). Previous studies have established roles for the mechanical microenvironment in regulating cells of the PRE lineage, facilitating differentiation of XEN cells into VE, EPI rosette formation, attachment of embryoid bodies to the substrate and derivation and differentiation of mouse and human ESCs (Cesare et al., 2022; Kohler et al., 2023; Kunath et al., 2005; Mole et al., 2021; Neagu et al., 2020; Paca et al., 2012). In our study, we identified a specific integrin and FN expression pattern in the PRE at E4.0-E4.5, which implicates ECM binding and its role in signalling for ICM lineage specification.

Itgβ1^−/−^, Itgα5^−/−^ and FN^−/−^ are all embryonic lethal genotypes (Carlson et al., 2008) and it has been shown that the PRE layer fails to resolve into a monolayer in Itgβ1^−/−^ mouse embryos and embryoid bodies (Kim et al., 2022; Liu et al., 2009; Moore et al., 2014; Stephens et al., 1995). Integrin function in the mouse embryo has been previously linked to adhesion, physical segregation or further differentiation of cell types rather than initial cell fate choices (Berger et al., 2003; Liu et al., 2009). However, some studies have demonstrated involvement of integrins in signalling pathways such as FGF/ERK with cell specification outcomes (Aguirre-Ghiso et al., 2003; Chaurasia et al., 2006; Fafilek et al., 2018; Hayashi et al., 2007). Moreover, mESCs grown on FN (and laminin) have been shown to induce integrins leading to an increase in pERK levels, while Itgβ1^−/−^ EBs revealed a decrease in pERK signal (Hayashi et al., 2007). This led us to investigate the potential role of ECM/integrin signalling in the initial PRE fate specification. Indeed, our results suggest that FN signalling through ITGα5β1 sensitises cells so that ERK phosphorylation can be induced by endogenous binding of FGF4 to its receptors, FGFR1 and FGFR2. The connection between FN binding to integrins and pERK activity we have uncovered suggests that the signalling role of ECM is important for ICM fate specification.

Having established FN and soft hydrogels as the preferred substrate, our NPRE derivation strategy focused on controlling NPRE cell maturation by modulating FGF4 concentration. We were unable to prevent our cells from progressing to more mature fates over successive passaging, regardless of the presence of FGF4, although we achieved contribution to the PRE lineage by injection into host blastocysts or aggregation with mESCs through multiple passages (up to P15). The variability in contribution and persistence of self-renewal potential between PRE cell lines may be attributable to the differential state of maturation between cells at the time of retrieval from the embryo (Grabarek et al., 2012). Early mouse embryo development is classified as ‘regulative’, meaning variable in length and timing of cell division, whereas embryos of most invertebrates are considered to be at least partially ‘mosaic’, undergoing a more rigid pattern of cleavage. Consistent with this, other lines, such as PRE stem cells, maintained morphology for up to five passages (Ohinata et al., 2022), while naïve endoderm cells, which correlated with E3.5 ICM cells immediately after differentiation, assumed an expression signature closer to XEN cells in mid and late passages (Anderson et al., 2017; Linneberg-Agerholm et al., 2024). Taking all studies together, it appears that the inability to capture NPRE cells in a steady state remains an obstacle to deriving immortal self-renewing PRE cells *in vitro*.

Our study adds mechanical and ECM adhesion information to the strategy of NPRE cell line derivation, but further research is needed to obtain homogeneous and stable cell lines. Given their initial correlation to the expression pattern of embryonic PRE cells and their continuing functionality over early passages, NPRE cells present a promising tool for *ex vivo* embryo studies. The developmental progression of the cells in culture offers the potential to study ICM specification into EPI and PRE *ex vivo*, using FGF− cells, or PRE differentiation from E4.0 to post-implantation fates, using FGF+ cells.

## MATERIALS AND METHODS

### Mouse husbandry

Mouse embryos were obtained from natural matings. Embryo staging was calculated on the assumption that copulation took place at midnight and therefore at noon the following day the embryos were assigned the stage embryonic day E0.5. Embryos were flushed using M2 (Sigma-Aldrich) from the oviducts (E2.5) or uterine horns (E3.5-E4.5). Mice were maintained on a lighting regime of light:dark 12:12 h with food and water supplied *ad libitum*. This study was regulated under the Animals (Scientific Procedures) Act 1986 Amendment Regulations 2012 following ethical review by the University of Cambridge Animal Welfare and Ethical Review Body (AWERB) under relevant Home Office licences (Project licences 80/2597 and P76777883). CD1 (wild type) and Pdgfrα^H2B-GFP/+^(F1) mice (Hamilton et al., 2003) were utilised in this study for cell line derivation; CD1 embryos were used as host for chimeric injections.

### Embryo treatments

Whole mouse blastocysts were treated with antibodies to inhibit integrin activity from E3.5 until the expanded blastocyst stage (∼E4.5). Embryos were cultured in 10 µM CD49e (BioLegend, 103817) to block the activity of integrin α5, and 10 µM CD29 (BioLegend, 102235) to block the activity of integrin β1. Control embryos were incubated in 10 µM Ctrl rat IgG2a (BioLegend, 400543) and 10 µM Ctrl Armenian hamster IgG (BioLegend, 400969). Additionally, embryos were treated with 25 µM ATN 161 (Bio-Techne, 6058) was used to simultaneously block ITGα5β1. E3.25 mouse early blastocysts were treated with 1 µg/ml FGF4. All embryos were cultured in drops of Blast (Origio, 83050010) or N2B27 (Table S2) under mineral oil (SAGE, ART4008) in a humidified incubator at 37°C, 5% CO_2_.

### scRNA-seq (embryos, cells)

RNA-seq samples were processed with bcbio v1.2.0 (https://doi.org/10.5281/zenodo.3564938). Reads were quality checked with FastQC v0.11.9 (https://www.bioinformatics.babraham.ac.uk/projects/fastqc) and reads quantified with Salmon v1.9.0 (Patro et al., 2017). STAR v2.6.1d (Dobin et al., 2013) was used to align the reads to the reference genome GRCm38, with duplicates marked by Picard MarkDuplicates v2.27.4 (https://github.com/broadinstitute/picard), and the alignments quality checked with BEDTools genomecov v2.30.0 (Quinlan and Hall, 2010), SAMtools stats v1.16 (Li et al., 2009), DupRadar v1.28.0: (Sayols et al., 2016), PreSeq v3.1.2 (Daley and Smith, 2013) and QualiMap v2.2.2 (Okonechnikov et al., 2016). Sample similarity was calculated with DESeq2 v1.28 (Love et al., 2014). Downstream analysis was performed in R v4.1.1. Samples with fewer than 4000 reads or fewer than 250 expressed genes were excluded, and embryo samples with fewer than 4000 expressed genes were excluded. Read counts were normalised to library sizes and log transformed. Principal component analysis was performed on embryonic PRE cells and cultured XEN cells using the 50 genes with the greatest coefficient of variance, and P0 NPRE cells were mapped onto this embedding. A curated list of PRE-, parietal endoderm- and VE-associated markers was used to generate expression heatmaps of embryonic PRE, P0 NPRE cells and XEN cells at passage 5.

### Immunostaining blastocyst and ICMs

Mouse blastocysts and isolated ICMs were fixed in 4% paraformaldehyde (PFA; Sigma-Aldrich) in PBS for 15 min at room temperature (RT) followed by a wash in PBS+3% polyvinylpyrrolidone (Sigma-Aldrich). Samples were permeabilised in PBS+3% polyvinylpyrrolidone+0.25% Triton X-100 (Sigma-Aldrich) for 15 min at RT and blocked in PBS+2% donkey serum (Sigma-Aldrich)+0.1% bovine serum albumin (BSA) (Sigma-Aldrich)+0.01% Tween20 (Sigma-Aldrich) for 30 min at RT. Primary and secondary antibodies were diluted in blocking buffer. Primary antibody incubation was performed overnight at 4°C followed by three 15 min washes in blocking buffer. Secondary antibody incubation, together with Hoechst 33342 (1:2000) for nuclear staining, was performed for 1-2 h at RT in the dark followed by three washes in blocking buffer for 15 min. Samples were imaged through a poly-D-lysine-coated Mattek dish (P356-0-14) while immersed in blocking buffer.

pERK staining using tyramide amplification was performed according to Azami et al. (2019). Briefly, mouse embryos were fixed in freshly prepared 8% PFA in PBS+pSTOP (Roche) for 10 min at RT. Blastocysts were then washed twice for 5 min each wash in PBS+0.1% Triton X-100 (PBST)+0.1% BSA at RT, prior to permeabilisation in PBS+0.5% Triton X-100+0.1% BSA for 5 min and blocking in PBST+0.1% BSA+10% donkey serum for 30 min at RT under agitation. Primary and secondary antibodies were diluted in blocking buffer. Samples were incubated overnight in primary antibodies, including pERK (Cell Signaling Technology, 4370P) at 4°C. The following day, embryos were washed in PBST+0.1% BSA for sequential washes of 5, 10, 15, 20 and 20 min at RT under agitation. Secondary antibody incubation, including donkey anti-rabbit-HRP (Jackson ImmunoResearch, 711-035-152), was performed at RT for 2 h in the dark. Embryos were then washed three times in PBS+0.1% Tween20 for 10 min. pERK-HRP signal was amplified using tyramide (Thermo Fisher Scientific, B40932) solution. Briefly, embryos were incubated in tyramide solution prepared following the manufacturer's specification for 15 min at RT prior to transfer into reaction stop reagent. Finally, embryos were washed in PBS+0.1% Tween20+0.1% BSA for 5 min before imaging. See Table S1 for details of primary and secondary antibodies.

### Immunostaining cells

NPRE cells on hydrogels or TCP coverslips (Thermo Fisher Scientific, 174950) were fixed with 4% PFA for 10 min at RT followed by two washes in PBS. Cells were permeabilised and blocked simultaneously in PBST for 2-4 h at RT. Primary antibody incubation was performed overnight at 4°C. The next day, cells were washed three times in PBST for 15 min prior to secondary antibody incubation for 1-2 h (with or without Hoechst for nuclear staining) at RT in the dark. Cells were then washed twice at RT with PBST with a final wash in PBS overnight followed by mounting using Vectashield (Vector Laboratories, H-1000-10).

### Immunosurgery and single-cell dissociation

The zona pellucida surrounding mouse blastocysts was removed using Acid Tyrode (Sigma-Aldrich, T1788) at RT. Mouse embryos were then incubated with anti-mouse antibody (Sigma-Aldrich, M5905) 1:5 in N2B27 (Table S2) for 1 h in the humidified incubator at 37°C, 5% CO_2_. Following three washes in N2B27, the blastocysts were treated with 1:5 rat serum (made in-house) in N2B27 for 45 min prior to incubation in N2B27 for 30 min to 1 h. During this time, the outer layer corresponding to the TE layer lysed and changed morphology. For complete removal of the TE, the embryo was taken through capillaries of sizes similar to that of the ICM.

### ICM aggregation

DN-09 needles (BLS) were used to create small cavities in the lid of IVF dishes (Falcon, 353653). Cavities were filled with the appropriate medium according to experiments and covered with mineral oil (SAGE, ART4008) prior to equilibration in a humidified incubator at 37°C, 5% CO_2_. Two to three isolated ICMs were carefully placed next to one another, in contact, into the cavities filled with media and immediately placed into the incubator for aggregation.

### StemBond hydrogel fabrication

Soft (3.5 kPa) and stiff (160 kPa) hydrogels were made according to Labouesse et al. (2021). Hydrogels were activated with the ECM of choice [FN (Sigma-Aldrich, FC010), laminin (Thermo Fisher Scientific, CC095)] and blocked in ethanolamine for 30 min at RT followed by three washes in PBS prior to being placed in tissue culture dishes. Hydrogels were stored at 4°C in PBS and equilibrated in a humidified 37°C, 5% CO_2_ incubator with basal medium for 2-12 h prior to cell seeding.

### NPRE derivation and culture

E3.5 Pdgfrα^HB2-GFP/+^ isolated mouse ICMs were incubated in Accutase (Stem Cell Technologies) for 10-15 min at 37°C prior to transfer to a drop of NPRE media (Table S3) to be dissociated into single cells using glass capillaries, then 50-70 single ICM cells were seeded on 4-well plates (Nunc™) either directly in contact with plastic or on top of soft hydrogels coated with the appropriate ECM (i.e. laminin, FN or mixture of both). The culture media recipe can be found in Table S3. Note that the Rock inhibitor (iRock; Y27632) was only used during passaging.

P0 NPRE colonies were passaged by first separating the colony from the hydrogel, then washing in PBS (Sigma-Aldrich) and incubating in 0.025% trypsin (Invitrogen) during 10-15 min followed by single-cell dissociation using glass capillaries. Single cells were immediately seeded onto new equilibrated hydrogels. Later passage NPRE colonies were passaged within the 4-well plate, following removal of NPRE media, washing with PBS and incubation in 400 µl trypsin for 10 min in the incubator. Colonies were separated from hydrogel and dissociated into single cells by exhaustive pipetting, in a laminar flow hood under a stereomicroscope. The cell mixture was centrifuged at 300 ***g*** for 3 min. The optimal cell density was considered to be 5×10^3^ per well in a 4-well plate. iRock was added to the cells after passaging to improve cell survival.

### Blastocyst injection

Pdgfrα^H2B-GFP/+^ NPRE cells were transfected with an mKO plasmid (kind gift from Masaki Kinoshita, University of Nottingham, UK). Five mKO-Pdgfrα^H2B-GFP/+^ NPRE cells were injected into (1) 8-cell morulae via a laser-generated hole in the zona just big enough for injection pipette to enter (Alexandrova et al., 2016; Poueymirou et al., 2007) or (2) E3.5 mouse blastocysts and cultured for 48 or 24 h, respectively, in a humidified incubator at 37°C and 5% CO_2_. Embryos were fixed at E4.5 in 4% PFA.

### Aggregate formation

ICM-like aggregates were formed by mixing 20 NPRE FGF+ cells with 15 mESCs, or by mixing 20 PRE-like NPRE FGF− cells with 15 EPI-like NPRE FGF− cells. To achieve this, NPRE FGF− cells were separated into EPI-like and PRE-like in a previous passage based on morphology and PDGFRA-GFP expression. All cells were washed in PBS and dissociated using 0.025% trypsin for 10 min at 37°C. The pellet was resuspended in N2B27 (Table S3)+10 ng/ml LIF+10 µM iRock. The cell number was counted using a haematocytometer and the density was adjusted to 15 or 20 cells in 50 µl depending on the cell type. Cells were seeded in non- adherent u-bottom 96-well plates (Greiner) containing 50 µl of pre-equilibrated N2B27 (Table S3)+10 ng/ml LIF+10 µM iRock and cultured in suspension in a humidified incubator at 37°C and 5% CO_2_. The aggregates were fixed after 8, 24 and 48 h in 4% PFA and immunostained in the same way as mouse blastocysts.

### Image acquisition, analysis and quantification

Embryos, cells and aggregates were imaged using a Leica Stellaris 8 confocal microscope or a ZEISS LSM980 confocal microscope. 3D confocal images of mouse embryos were segmented using MINS software based on the nuclei channel. Following single-cell segmentation, MINS provided quantification of the nuclear fluorescence intensity of each transcription factor in each individual cell across the entire embryo. We created histograms using data from E4.5 embryos to find the expression threshold separating TE from ICM, and ICM into EPI and PRE cells. The threshold value was used to separate populations and count the cell number per cell type. The Fiji plug-in ‘Cell Counter’ was used to corroborate the cell counts provided by MINS analysis. Chimera contribution was counted using the Fiji plug-in ‘Cell Counter’. Colony area was measured using Fiji. Data analysis and presentation was performed in R, scatter and violin plots were created using the function ‘ggplot2’ and histograms using ‘ggExtra’. Statistical analysis was also performed in R; all the statistical analysis in this paper were two-tailed Student's *t*-tests.

## Supplementary Material



10.1242/develop.205226_sup1 Supplementary information
