# Supplementary material for: Harnessing the murine inner cell mass mechanical environment enhances derivation of in vitro nascent primitive endoderm precursor cells
Source: Development. 2026 Jun 11;153(16):dev205226. doi: 10.1242/dev.205226 (PMC13286370; doi:10.1242/dev.205226)
Supplement: Supplementary information [file develop-153-205226-s1.pdf]

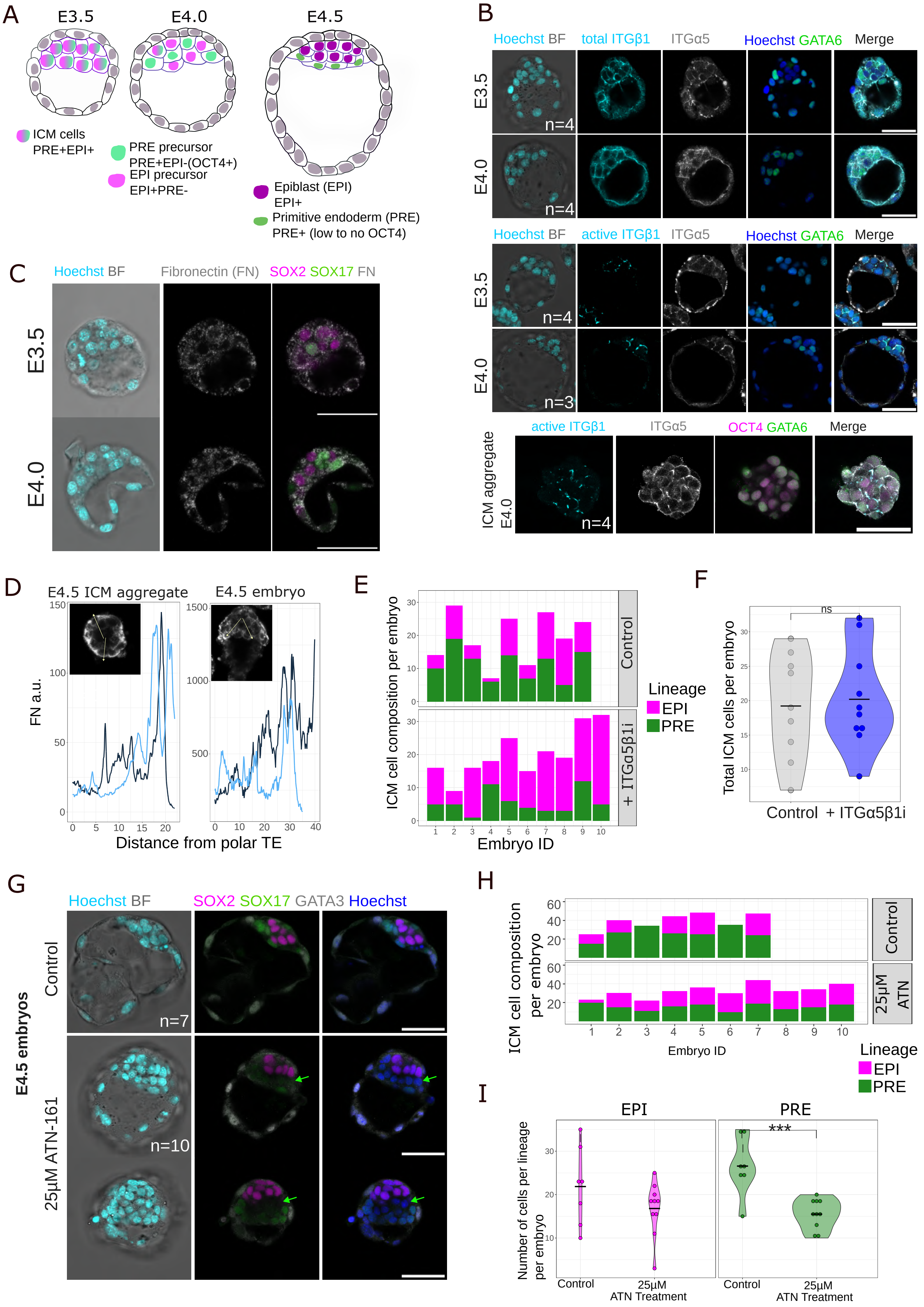

Development • Supplementary information

Fig. S1. No difference in integrins or fibronectin protein expression and activity before E4.5. A. Diagram showing pre-implantation blastocyst from E3.5 to E4.5 as the ICM differentiates into EPI and PRE. B. Representative confocal image of mouse embryos at E3.5 and E4.0 immunostained for total ITGβ1 (top panel; cyan) and active ITGβ1 (middle and bottom panel; cyan), ITGα5 (grey) and GATA6 (green) and Oct4 (magenta), bottom panel. Nucleus is shown with Hoechst. C. Representative confocal images of E3.5 and E4.0 mouse blastocysts immunostained for Fibronectin (grey), SOX2 (magenta) and SOX17 (green) and nucleus shown with Hoechst. D. Representative intensity profile of fibronectin (FN) from centre EPI cells into outer PRE cells in the E4.5 ICM aggregate, and from polar TE across EPI into PRE in the E4.5 embryo, marked as lines on the confocal images; arrowheads indicate the end of measurement. E. Stacked bars plot quantifying the number of cells belonging to EPI or PRE lineage per embryo in the control, or in the presence of integrin blocking antibodies. F. Violin plot showing the average total cell number in control and embryos treated with antibodies to block ITGα5β1 activity. Statistical significance was assessed using a t-test (* $p < 0.05$) G. Representative confocal image of mouse embryos treated with ATN-161 compared to the untreated control (top panel). Embryos were immunostained for SOX2 (magenta, EPI), SOX17 (green, PRE) and GATA3 (grey, TE). The nucleus is shown with Hoechst. Green arrows indicate ICM cells negative for SOX2 and SOX17. Scale bar is 50μm in all images. H. Stacked bar plot quantifying the number of cells belonging to EPI or PRE per embryo in the control or when cultured in the presence of ATN-161. I. Violin plot quantifying the average number of cells belonging to each lineage (EPI or PRE) per embryo in control and treated embryos. Statistical significance was assessed using a t-test (*** $p < 0.0005$).

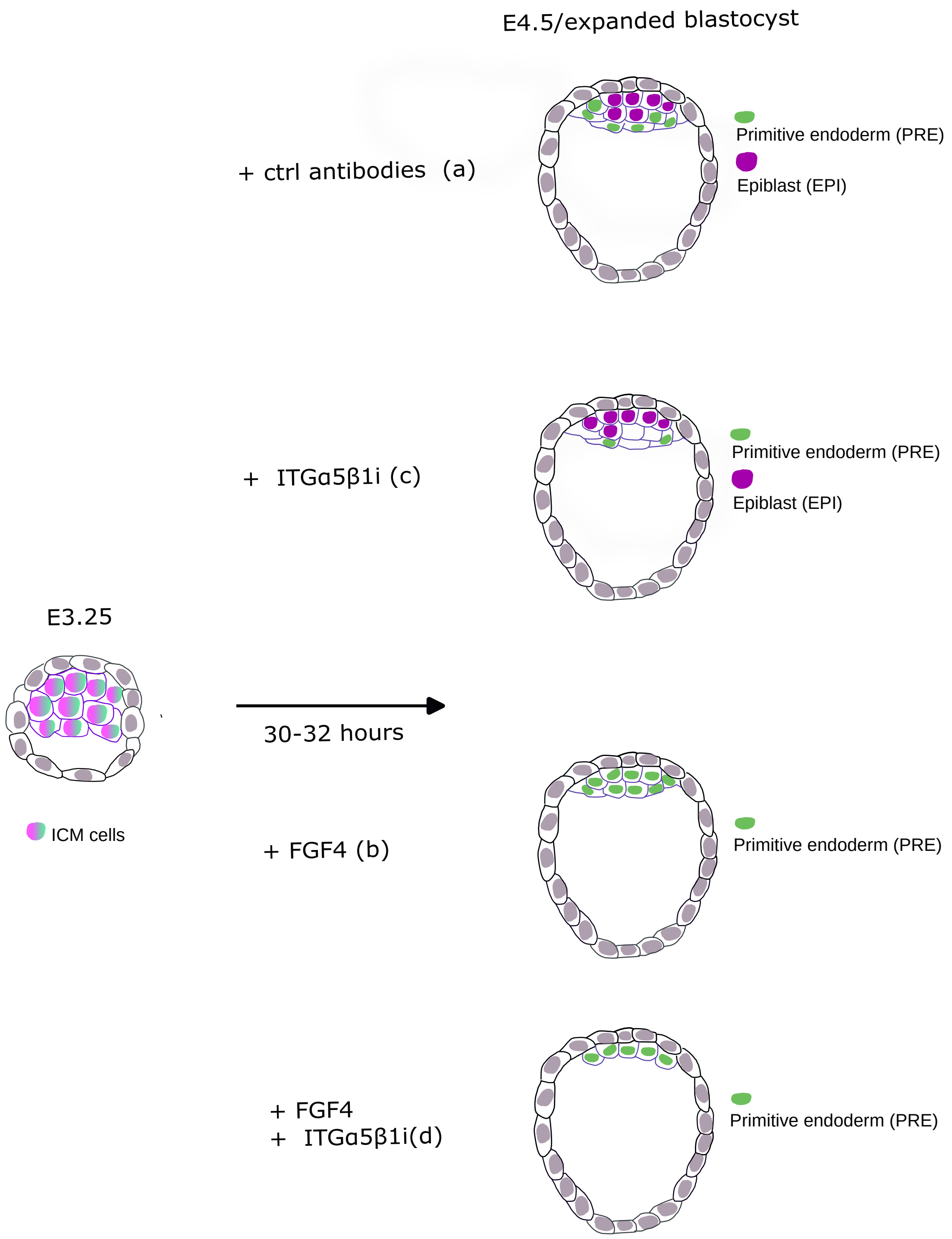

Fig. S2. Schematic experimental diagram of FGF4 and ITGβ1α5 blocking antibodies on mouse early blastocysts.

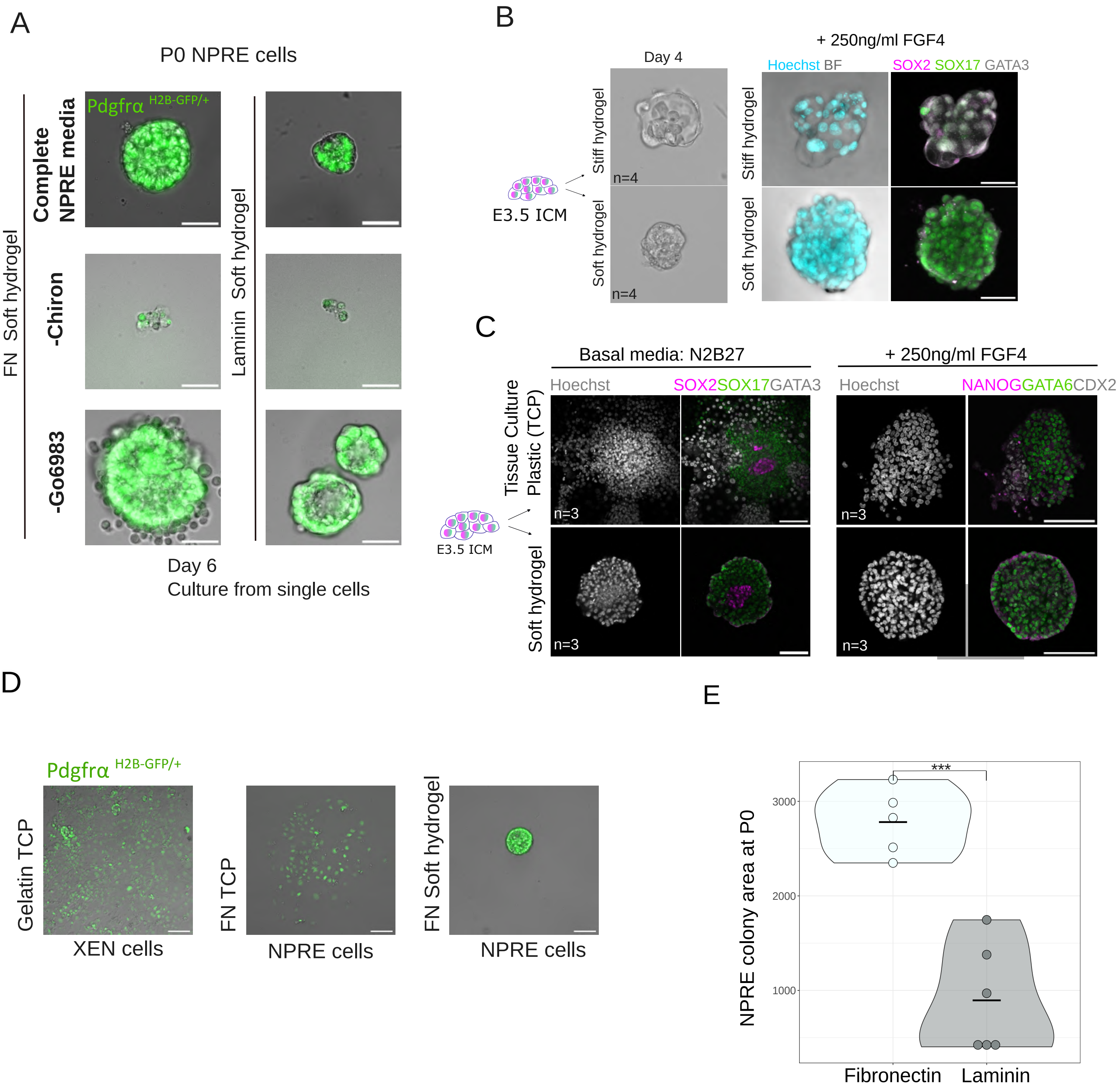

Fig. S3. Soft substrates promote PRE lineage over TE. A. Day 6 Pdgfrα $^{H2B\text{-}GFP/+}$ cells grown on FN-coated soft hydrogels in complete NPRE media (top panel), NPRE media without Chiron (middle panel) or without Gö6983 (bottom panel). B. Representative brightfield and confocal images of E3.5 mouse ICM outgrowth after 4 days treatment with FGF4 on stiff hydrogels (top panel) or soft hydrogels (bottom panel). Outgrowths were immunostained for SOX2 (magenta), SOX17 (green), GATA3 (grey). C. Representative confocal images of E3.5 mouse ICM outgrowths after 4 days culture in basal media (left panel) or treated with FGF4 (right panel) on tissue culture plastic (TCP) (top panel) or soft hydrogel substrates (bottom panel). Outgrowths were immunostained for EPI markers SOX2/NANOG (magenta), PRE markers SOX17/GATA6 (green) and GATA3/CDX2 (grey). The nucleus is shown with Hoechst. D. Representative brightfield images of Pdgfrα $^{H2B\text{-}GFP/+}$ cells grown in XEN media (top), NPRE media on TCP (middle), or NPRE media on soft hydrogels coated with FN (bottom). Derivation on FN TCP for FGF- cells was performed twice, and for FGF+ 3 times. Scale bar is 100 μm in all images. E. Violin plot quantifying the colony area of NPRE cells seeded on FN-coated hydrogels or Laminin coated hydrogels. Statistical significance was assessed using a t-test (*** $p < 0.0005$).

A

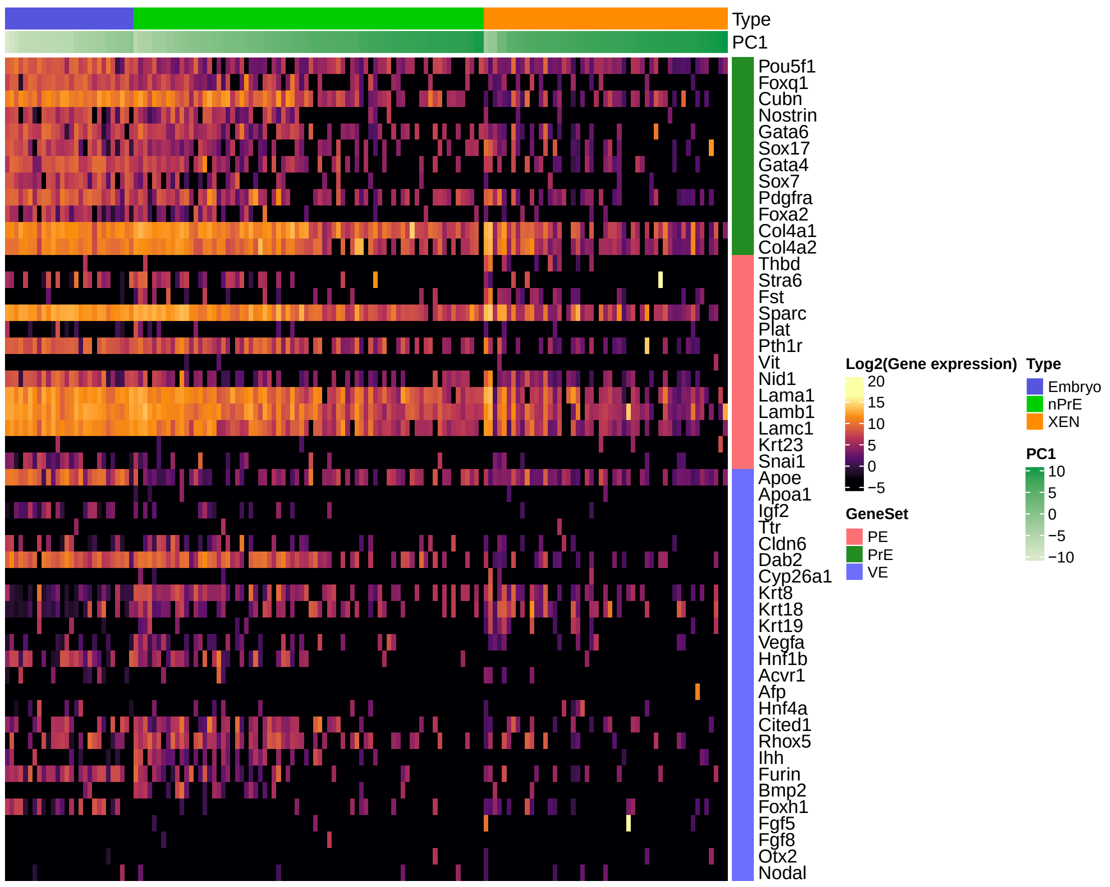

B

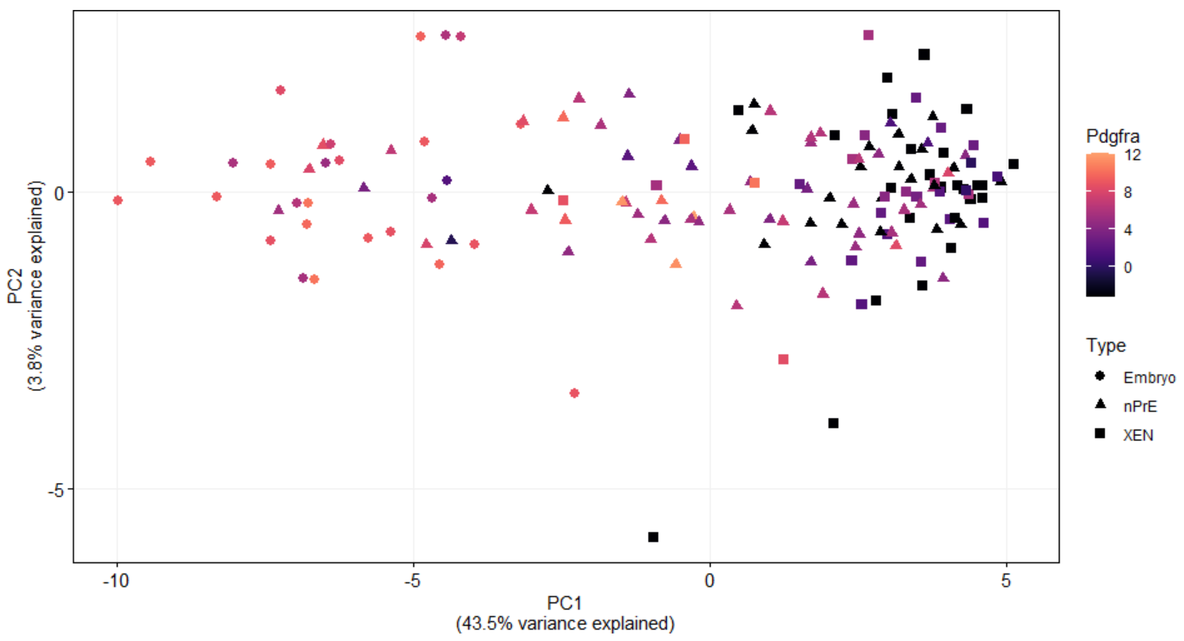

Fig. S4. Gene expression in NPRE cells compared to pre-implantation and post-implantation PRE. A. Heatmap of single cell RNA seq for embryonic PRE, parietal endoderm (PE) and visceral endoderm (VE). Normalized gene expression values are represented by a colour heat map spectrum from high expression (yellow) to low expression (black). Data shown are biological replicates of E4.5 embryonic PRE cells, PO NPRE cells and XEN cells. B. Principal component analysis (PCA) plot of E4.5 PRE embryonic cells, NPRE cells, and XEN cells coloured according to expression level of Pdgfrα. Each dot represents a single cell.

Development • Supplementary information

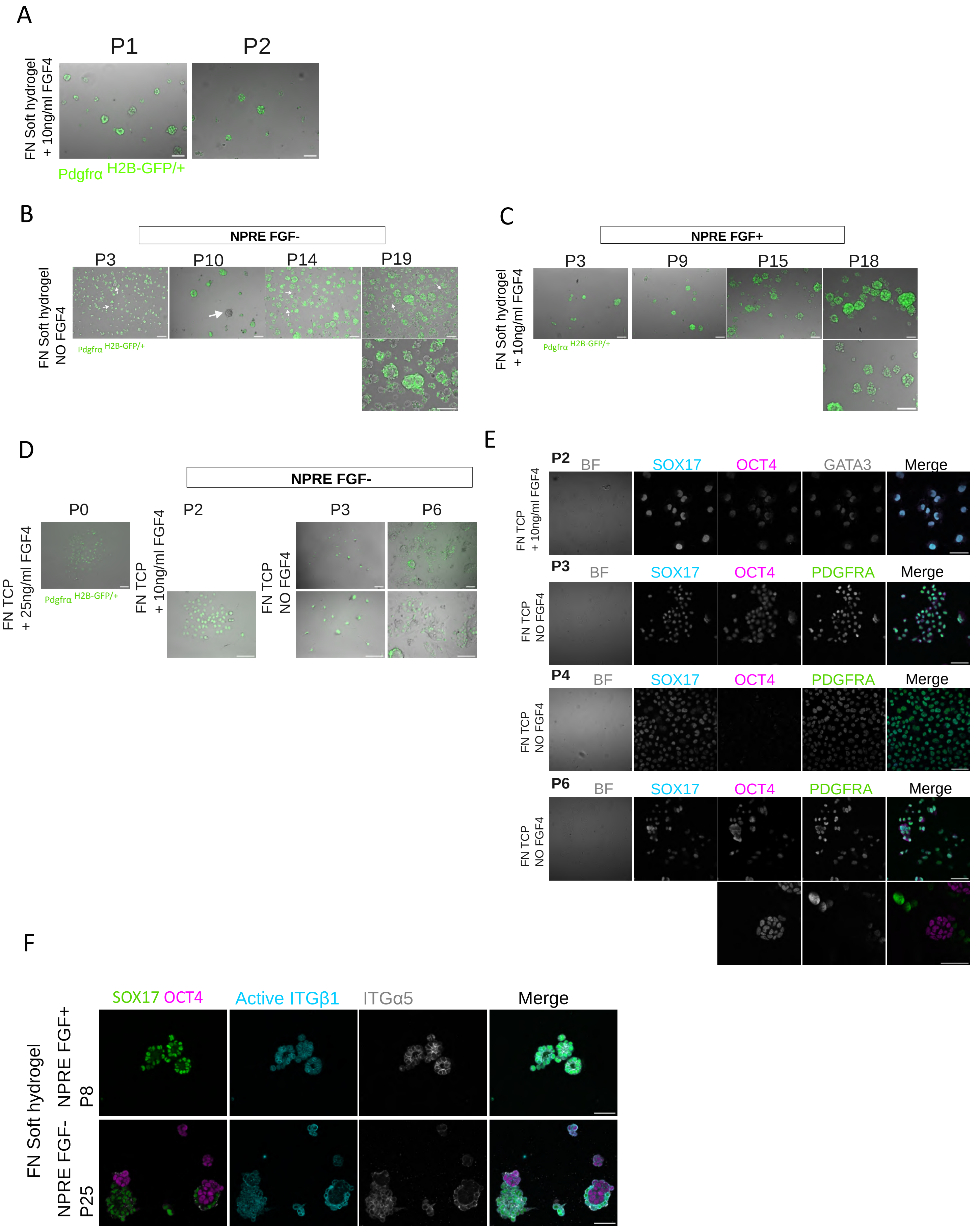
A
P1
P2
FN Soft hydrogel + 10ng/ml FGF4
Pdgfrα H2B-GFP/+
B
NPRE FGF-
P3
P10
P14
P19
FN Soft hydrogel NO FGF4
Pdgfrα H2B-GFP/+
C
NPRE FGF+
P3
P9
P15
P18
FN Soft hydrogel + 10ng/ml FGF4
Pdgfrα H2B-GFP/+
D
NPRE FGF-
P0
P2
P3
P6
FN TCP + 25ng/ml FGF4
Pdgfrα H2B-GFP/+
FN TCP + 10ng/ml FGF4
FN TCP NO FGF4
E
P2
BF
SOX17
OCT4
GATA3
Merge
FN TCP + 10ng/ml FGF4
P3
BF
SOX17
OCT4
PDGFRA
Merge
FN TCP NO FGF4
P4
BF
SOX17
OCT4
PDGFRA
Merge
FN TCP NO FGF4
P6
BF
SOX17
OCT4
PDGFRA
Merge
FN TCP NO FGF4
F
SOX17 OCT4
Active ITGβ1
ITGα5
Merge
FN Soft hydrogel
NPRE FGF+ P8
NPRE FGF- P25

Fig. S5. NPRE maintenance over passages is dependent on the concentration of FGF4 and independent of the substrate. A. Overlay of brightfield and Pdgfrα $^{H2B\text{-}GFP/+}$ images at P1 and P2 of NPRE derivation. B.C. Overlay of brightfield and Pdgfrα $^{H2B\text{-}GFP/+}$ images throughout the time-course of NPRE derivation in the absence (B) or presence of FGF4 (C). A minimum of 5 colonies were imaged per passage per ECM. D. Overlay of brightfield and Pdgfrα $^{H2B\text{-}GFP/+}$ showing PRE cells from P0 to P6 grown on TCP coated with fibronectin (FN). FGF4 concentration is first decreased before being completely removed from the media. Derivation on FN TCP for FGF- cells was performed twice, and for FGF+ 3 times. E. Representative confocal images of NPRE cells maintained on FN-coated TCP from P2 to P6, following the removal of FGF4 from the media at P3. Cells are immunostained for SOX17 (cyan), OCT4 (magenta), PDGFRA (green) and GATA3 (grey). F. Representative confocal image of P8 FGF+NPRE cells (top panel) and P25 FGF-NPRE cells (bottom panel) immunostained for SOX17 (green), OCT4 (magenta), active ITGβ1 (cyan) and ITGα5 (grey) cultured on FN-coated soft hydrogels. Scale bar is 50 μm in all images.

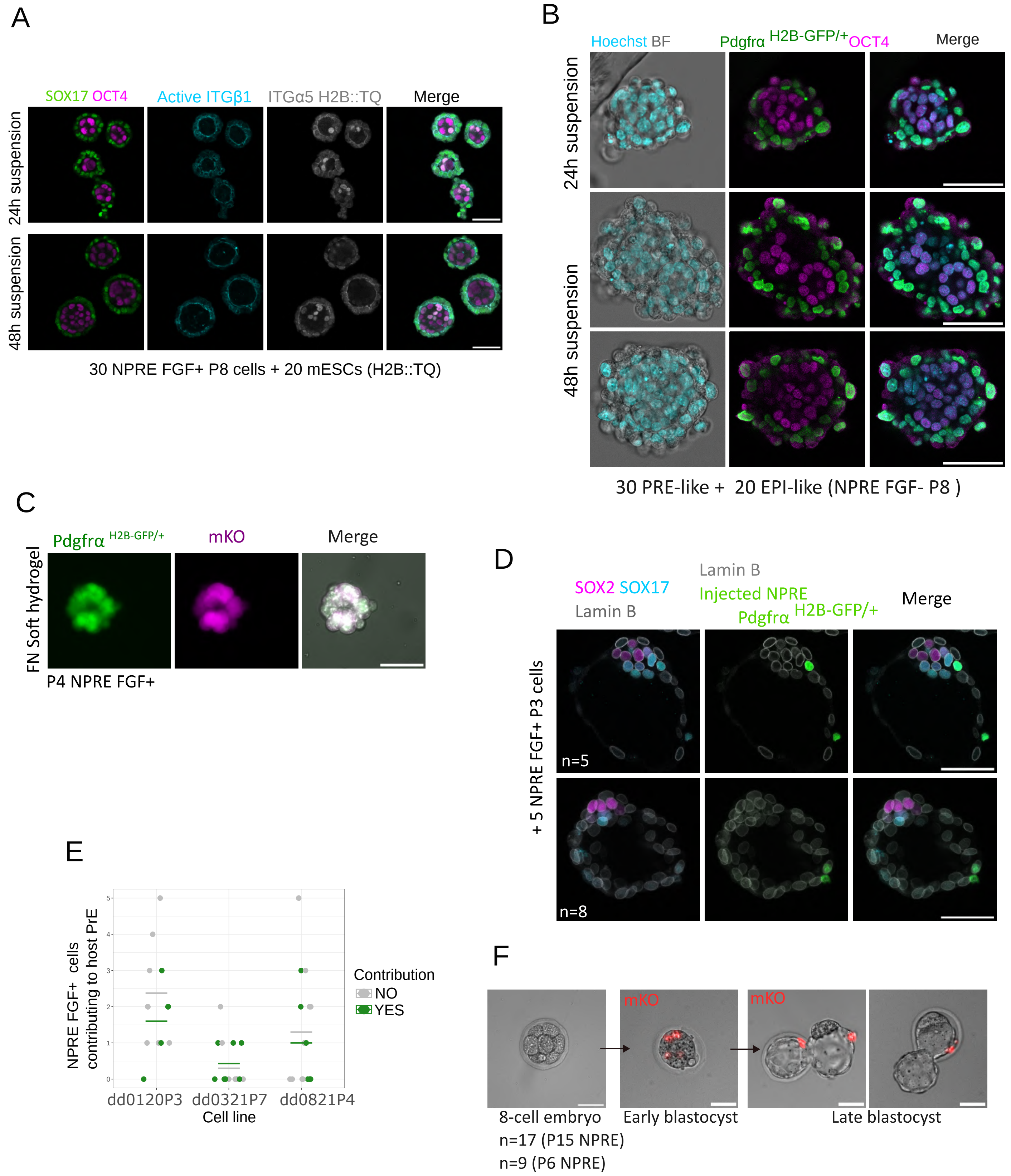

Fig. S6. NPRE aggregates reproduce the integrin expression pattern observed in mouse blastocysts. A Representative confocal image of ICM aggregates made of 30 FGF+NPRE cells and 20 mESCs following 24h and 48h culture in suspension. mESCs were heterogeneously expressing H2B::TQ (grey). ICM aggregates were immunostained for SOX17 (green), OCT4 (magenta), active ITGβ1 (cyan) and ITGα5 (grey). B. Representative confocal images of ICM aggregates made of FGF4-NPRE cells following initial separation of EPI-like and PRE-like prior to mixing 30 PRE-like and 20 Epi-like. Aggregates were maintained in culture 24 and 48 h and immunostained for OCT4 (magenta). The nucleus is shown with Hoechst. C. NPRE Pdgfrα $^{H2B\text{-}GFP/+}$ cells transfected with mKO (magenta) at P4 were used for injection in Figure 6C. D. Representative confocal images of host embryos showing integrated NPRE cells in the ICM (middle panel) or NPRE adherence to the TE. E. Scatter plot quantifying the contribution of NPRE cells to the host PRE in additional datasets comprising different passage number. F. Brightfield/mKO overlay images following NPRE cells (mKO, red) at P3, injected at the 8-cell stage until fixation of host embryo at E4.5. Scale bar is 50 µm in all images.

Development • Supplementary information

Table S1. Primary and secondary antibody list

Antibody (host)	Dilution	Supplier	Catalogue no
GATA6 (goat)	1:200	R&D	AF1700
GATA6 (rabbit)	1:200	Cell Signaling Technology	5851S
SOX17 (goat)	1:200	R&D	AF1924
SOX7 (goat)	1:200	R&D	AF2766
GFP (chicken)	1:200	Thermo Fisher	A10262
OCT4 (C-10) (mouse)	1:200	Santa Cruz	SC-5279
SOX2 (rat)	1:200	eBioscience	14-9811-80
NANOG (rat)	1:200	eBioscience	14-5761-80
Fibronectin (goat)	1:100	Rockland	600-401-117-0.1
Total integrin β1	1:200	SIGMA	MAB1997
Active integrin β1 (BD9E67)	1:100	BDPharmigen	553715
Integrin α5	1:100	ABCAM	ab150361
pERK	1:50	Cell Signaling Technology	4970P
GATA3	1:300	ABCAM	ab199428
CDX2	1:200	ABCAM	ab157524
Vimentin (rabbit) D21H3	1:100	Cell Signaling Technology	5471T
E-cadherin (rat)	1:100	SIGMA	U3254

Antibody (host)	Dilution embryos/cells	Supplier	Catalogue no
Hoechst 33342	1:500/1:1000	Thermo Fisher	62249
Donkey anti-goat-405	1:500/1:1000	Abcam	ab175664
Donkeyanti-rabbit-405	1:500/1:1000	Abcam	ab175651
Goat anti-chicken-488	1:500/1:1000	Thermo Fisher	A11039
Donkey anti-goat-488	1:500/1:1000	Thermo Fisher	A32814
Donkey anti-rabbit-555	1:500/1:1000	Thermo Fisher	A31572
Donkey anti-mouse-555	1:500/1:1000	Thermo Fisher	A31570
Chicken anti-rat-647	1:500/1:1000	Thermo Fisher	A21472
Donkey anti-mouse-647	1:500/1:1000	Thermo Fisher	A31571

Development • Supplementary information

Table S2. N2B27 medium composition

Components	Source	Cat no	Final concentration
DMEM/F12	Thermo Fisher	21331-020	
Neurobasal	Thermo Fisher	21103049	
B27 (50x)	Invitrogen	17504044	0.5x
N2	Made in house		1x
β-mercaptoethanol (50mM)	Thermo Fisher	31350-010	50µM
L-glutamine (200mM)	Thermo Fisher	25030081	2mM

Table S3. Nascent PrE medium composition at P0

Components	Source	Cat no	Final concentration
RPMI1640	Sigma	R8758	
FBS	GE Healthcare Life Sciences	SV30160.02	10%
β-mercaptoethanol (50mM)	Thermo Fisher	31350-010	50µM
Sodium Pyruvate	Sigma	S8636	0.1%
LIF	Made in house		10 ng/ml
CHIR99021	abcr	AB253776	3µM
PDGF-AA	eBioscience	24-8989-80	10ng/ml
Go6983 (iAPKC)	Bio-Techne	2285	2µM
FGF4	Peprotech	100-31	25ng/ml
Heparin	Stem Cell Technologies	07980	1µg/ml
Y-27632 (iRock) (only added after passage)	Sigma/Merck	688000-100MG	10µM

Development • Supplementary information
