# Supplementary material for: Harnessing the murine inner cell mass mechanical environment enhances derivation of in vitro nascent primitive endoderm precursor cells
Source: Development. 2026 Jun 11;153(16):dev205226. doi: 10.1242/dev.205226 (PMC13286370; doi:10.1242/dev.205226)
Supplement: Supplementary file 1 [file develop-153-205226_review_history.pdf]

Harnessing the murine inner cell mass mechanical environment enhances derivation of *in vitro* nascent primitive endoderm precursor cells

Elena Corujo-Simon, Siiri I. Salomaa, Lawrence E. Bates, Ayaka Yanagida, William Mansfield, Hannes Becher, Jennifer Nichols and Kevin J. Chalut
DOI: 10.1242/dev.205226

Editor: Matthias Lutolf

Review timeline

Original submission:	9 September 2025
Editorial decision:	7 November 2025
First revision received:	19 March 2026
Editorial decision:	13 April 2026
Second revision received:	27 April 2026
Accepted:	12 May 2026

Original submission

First decision letter

MS ID#: dev.205226

MS TITLE: Harnessing the murine inner cell mass mechanical environment enhances derivation of in vitro nascent primitive endoderm precursor cells

AUTHORS: Elena Corujo-Simon; Siiri Salomaa; Lawrence E. Bates; Ayaka Yanagida; William Mansfield; Hannes Becher; Jennifer Nichols; Kevin J. Chalut

Dear Dr Nichols,

I have now received all the referees' reports on the above manuscript, and have reached a decision. The referees' comments are appended below.

As you will see, the referees express considerable interest in your work, but have some significant criticisms and recommend a substantial revision of your manuscript before we can consider publication. If you are able to revise the manuscript along the lines suggested, which may involve further experiments, I will be happy receive a revised version of the manuscript. Your revised paper will be re-reviewed by one or more of the original referees, and acceptance of your manuscript will depend on your addressing satisfactorily the reviewers' major concerns. Please also note that Development will normally permit only one round of major revision. If it would be helpful, you are welcome to contact us to discuss your revision in greater detail. Please send us a point-by-point response indicating your plans for addressing the referees' comments, and we will look over this and provide further guidance.

Please attend to all of the reviewers' comments and ensure that you upload both a 'clean' version of your Word file, along with a highlighted version clearly showing where you have made changes in the revised manuscript. Please avoid using 'Tracked changes' in Word files as these are lost in PDF conversion. I should be grateful if you would also provide a point-by-point response detailing how

© 2026. Published by The Company of Biologists under the terms of the Creative Commons Attribution License (https://creativecommons.org/licenses/by/4.0/).

you have dealt with the points raised by the reviewers in the 'Response to Reviewers' box. If you do not agree with any of their criticisms or suggestions please explain clearly why this is so.

Reviewer 1

This study by Corujo-Simon et al. addresses the persistent challenge of deriving stable in vitro cell lines representing the nascent primitive endoderm (PRE) of the pre-implantation mouse embryo, a critical component missing from current blastoid models. The authors claim that recapitulating the mechanical microenvironment of the inner cell mass (ICM) is a crucial factor for successfully capturing this transient cell state. They found that integrin α5β1-fibronectin signaling is linked to the FGF/ERK pathway, important for PRE specification. Using soft, fibronectin-coated hydrogels, they derived NPRE cell lines from E3.5 ICMs. These NPRE cells initially co-expressed ICM and PRE markers, consistent with an E4.0-E4.5 identity. Though not stable, these cells were functional, sorting correctly with mESCs and contributing to host blastocyst PRE lineages. The study concludes that mechanical and ECM cues enhance PRE-like cell derivation, offering a valuable system for studying early lineage decisions despite stability challenges.

Major comments:

Figure 2
- Figure 2D quantifies pERK signaling using a binary approach (pERK-positive vs. pERK-negative cells). A more rigorous, quantitative, and sensitive analysis could be achieved by measuring the mean fluorescence intensity of nuclear pERK on a per-cell basis, which might reveal more subtle inhibitor effects.
- If, as the authors propose, the primary effect of integrin inhibition is the downregulation of ERK signaling, then constitutively activating the pathway downstream of the integrin-receptor complex should rescue the PRE specification defect. For example an experiment treating integrin α5β1-inhibited embryos with a MEK activator could provide some insight for this causal link?

Figure 3
- In Figure 3A and within the text (method section), substrate stiffness should be stated instead of just soft or stiff. This is crucial for the reproducibility of the work and for understanding the specific mechanical window that supports NPRE cell derivation.
- In Figure 3D, the violin plot shows no statistically significant difference in colony area between fibronectin and fibronectin/laminin coatings, which appears to contradict the claim that fibronectin is superior based on the number and size of colonies. This discrepancy should be addressed; perhaps quantifying colony number in addition to area?

Figure 5
- In Figure 5C, it seems that there are more Nanog+ cells in P2 NPRE cells. Do these EPI-like cells arise from the de-differentiation of specified PRE precursors, demonstrating a remarkable degree of plasticity, or do they emerge from the selective expansion of a minor, undetected EPI-like population that persisted from the initial P0 culture? For example using a Sox17-CreERT2 driver to label PRE-like cells at an early passage and then tracking their fate after FGF removal would definitively distinguish between de-differentiation and selection of a pre-existing sub-population.

Figure 6
- The scatter plot in Figure 6E shows high variability in the number of contributing cells per embryo, ranging from a single cell to more than ten. The source of this variability is not discussed but could be linked to the inherent heterogeneity of the NPRE population?

Clonality The current derivation protocol starts from a population of 50-70 dissociated ICM cells, leading to a heterogeneous P0 population. Attempting to derive clonal NPRE lines from single cells would be highly informative. This would reveal whether the observed instability is an intrinsic property of all NPRE cells or if stable clones can be isolated from a heterogeneous starting pool.

Some current blastoid models are relying on mESCs. Could the finding of this manuscript be applied to sorted PRE cells from blastoids to derive a more nascent population of PREs?

© 2026. Published by The Company of Biologists under the terms of the Creative Commons Attribution License (https://creativecommons.org/licenses/by/4.0/).

Minor comments:

- the authors' claim of "reproducing the mechanical environment of the in vivo peri-implantation equivalent", may be an overstatement. The in vivo ICM is a highly complex, three-dimensional aggregate of cells with dynamic cell-cell and cell-ECM interactions. The authors' 2D hydrogel system, while a significant improvement over rigid plastic, is a simplification of this intricate niche. The language throughout the manuscript should be moderated to present the system as a step towards recapitulating the in vivo environment, rather than a faithful replica of it.

- Figure 1B: Integrin gene expression from single ICM cells (EPI and PRE precursors) at E4.0. The number of embryos needs to be specified.
Different PRE markers have been used even within one figure throughout the manuscript. Ex. Sup Fig 3C, Fig. 5D,E. Is that to diversify the markers? It would be better to include both rather than alternating between those.

- Consider including the expression of other ECM proteins, such as laminin, in Figure 1, particularly since it is further examined in Figure 3.

- In section : Integrin activity affects lineage acquisition via FGF/ERK signalling, the data demonstrate a requirement for integrin signaling for proper ERK activation, but do not exclude other potential mechanisms, such as integrin-mediated clustering of FGF receptors or regulation of receptor trafficking. The text should reflect this ambiguity.

- The abstract and introduction make claims about the potential for NPRE cells to integrate into blastoids. However, the experiments presented only show the formation of ICM-like aggregates with mESCs (Fig. 6A), not full blastoids that include a trophectoderm layer. The claims in the manuscript should be revised to more accurately reflect the data that is actually presented.

Reviewer 2

SUMMARY OF THE ADVANCE MADE IN THIS PAPER AND ITS POTENTIAL SIGNIFICANCE TO THE FIELD

In recent years, several stem cell-based models of early mammalian development have been reported. Although such models possess considerable potential for advancing research within the scientific community, particularly in the investigation of human preimplantation and early postimplantation development, several key challenges must be addressed to facilitate their successful application. One of the most important is the assurance that the models can fatefully replicate the physiology and behaviour of their in vivo counterparts. This specific issue was addressed by Chalut and colleagues in their current manuscript. Namely, the authors aim was to derive cells in culture that are equivalent of pre-implantation primitive endoderm (PRE). PRE is one of the 3 key lineages that arise early in development and so far, the attempts to capture preimplantation PRE state were not fully successful.
To achieve their goal, Chalut and colleagues' approach was to re-create in the cell culture the condition equivalent to those found in the ICM of the developing embryo. By testing various soft and stiff substrates in combination with most suitable extracellular matrix elements the authors were able to find the mechano-environment that supports derivation of PRE equivalent population.

The protocols and new information about the combined effects of integrin and mechano-environment on PRE and Epi maintenance included in this manuscript can be potentially very useful for the scientific community working on cell-based models of early mammalian development. However, several issues need to be addressed to help the reader to take full advantages of the data provided here.

Major issues

1. There is no clear explanation why the authors chose to specifically look at integrins but no other elements of ECM?

© 2026. Published by The Company of Biologists under the terms of the Creative Commons Attribution License (https://creativecommons.org/licenses/by/4.0/).

2. In the first section of the Results (Integrin α5β1 activity is necessary for PRE cell fate specification), the authors wrote: 'At E4.5, the difference in intensity of ITGα5 became more evident, and active ITGβ1 was clearly localized to the boundary between EPI and PRE' - can the authors provide some kind of measurements (plus nr of embryos/cells analysed) to support their claim?

3. Similarly, in the same section of the Results, the sentence 'However, as with the expression pattern of ITGα5, fibronectin expression was clearly localized in the sorted PRE beside the blastocoel in E4.5 embryos and outer cells of E4.5 ICM aggregates' also needs to be supported by measurements (plus nr of embryos/cells analysed).

4. The measurements should also be provided for the data currently presented in Fig 1E. (sentence 'Clear localisation of fibronectin in PRE cells at E4.5 was lost when combined with ITGα5β1 inhibition, remaining ubiquitously expressed in the ICM (Fig. 1E)'.

5. Conclusion from figure 1 and 2 is basically the same in the main text. Could the authors re-write this part to highlight what exactly is the main message from the data include in fig 2?

6. What was the timing in experiments presented in fig S2?

7. What was the reason for 'a significant decrease in the number of PRE cells in embryos subjected to integrin inhibition' (data presented in Fig. 2A-B)? Was it due to failure of specification or for example due to poor survival of PRE cells in such conditions?

8. The presentation of Fig 2B not easy to understand. What do the different boxes in y-axis represent? This should be explained better in the figure legend.

9. In the part of the Results 'Soft FN-coated substrates promote PRE growth' the authors mentioned that Lif removal resulted in poor survival and cited fig S3A but this figure does not have data on Lif removal.

10. In the part of the Results 'Soft FN-coated substrates promote PRE growth' the authors stated: 'Under FGF4 treatment, ICM outgrowths on soft hydrogels were enriched with PRE, while those on TCP also contained a TE population (Fig. S3C).' How was the enrichment calculated (fig S3C only contains representative images)?

11. From fig 5 B - it is not that clear to me which passage represent which stage of in vivo development. Could the authors provide a common timescale (with embryonic days (E) clearly marked on the timeline) for in vivo and in vitro (passages) and add it to the figure? Matching the exact passage to the desirable developmental stage will be important if the NPRE cells were to be used to create more sophisticated cell-based models of mammalian pre and peri-implantation development. It would also be good to discuss in the main text why while the progression of developmental stages is preserved in the subsequent passages, the length of the time for each in vivo and in vitro stage varies.

12. The way the figure description is written/organised in the current version of the manuscript is often very confusing. For consistency, all the figures legend should be structured in the same way for the same type of experiments/treatments. Also, all necessary information needs to be included.

* For example, panel F in Suppl fig 5 - the figure legend is not consistent with the panel D and E descriptions. Namely, it does not include information about the surface type and passage nr as provided in panels D and E. Why?
* Fig 6A - in the figure legend PRE cells are mentioned (which may indicate PRE cells isolated from ICM), whereas in the figure itself they are named NPRE - which suggests that they are the culture derived cells.
* Fig S6C description is very vague
* How many embryos were injected with NPRE cells in experiments described in FigS6F?

13. In the part titled "NPRE FGF+ cells can contribute to host blastocyst PRE and aggregate with mESCs" the authors wrote: "(…) we separated the cells based on morphology and cultured them as two distinct cell lines for one passage prior to aggregation" - more details is needed (like what were

© 2026. Published by The Company of Biologists under the terms of the Creative Commons Attribution License (https://creativecommons.org/licenses/by/4.0/).

the morphological criteria, were the cultures homogenous after separation, how this homogeneity was assessed etc.)

14. In the Aggregate formation part of MM it is described that NPRE FGF+ cells were mixed with ESCs in different proportions than NPRE FGF- . Can the authors provide the rationale why?

Minor points

1. The reasons for adding ICM aggregate data to fig 1 becomes apparent only after reading the whole manuscript. The authors may want to consider whether fig 1 is the best place for those data.

2. Fig 1G can be moved to supplementary figures as it does not necessarily add more info than fig 1H

First revision

Author response to reviewers' comments

Reviewer 1: This study by Corujo-Simon et al. addresses the persistent challenge of deriving stable in vitro cell lines representing the nascent primitive endoderm (PRE) of the pre-implantation mouse embryo, a critical component missing from current blastoid models. The authors claim that recapitulating the mechanical microenvironment of the inner cell mass (ICM) is a crucial factor for successfully capturing this transient cell state. They found that integrin α5β1-fibronectin signaling is linked to the FGF/ERK pathway, important for PRE specification.
Using soft, fibronectin-coated hydrogels, they derived NPRE cell lines from E3.5 ICMs. These NPRE cells initially co-expressed ICM and PRE markers, consistent with an E4.0-E4.5 identity. Though not stable, these cells were functional, sorting correctly with mESCs and contributing to host blastocyst PRE lineages. The study concludes that mechanical and ECM cues enhance PRE-like cell derivation, offering a valuable system for studying early lineage decisions despite stability challenges.

We thank the reviewer for this positive and accurate summary of our work, and hope that our revised manuscript clarifies their questions.

Major comments:

Figure 2
- Figure 2D quantifies pERK signaling using a binary approach (pERK-positive vs. pERK-negative cells). A more rigorous, quantitative, and sensitive analysis could be achieved by measuring the mean fluorescence intensity of nuclear pERK on a per-cell basis, which might reveal more subtle inhibitor effects.

We agree with the reviewer that a more rigorous, quantitative approach would allow us to reveal more subtle inhibitor effects. However, since pERK protein is frequently cytoplasmic, as apparent in Figure 2D, it is difficult to quantify levels of fluorescence on a cell-by-cell basis. Our aim was not to quantify the exact amount of pERK per cell; this would be very complicated, since within the blastocyst, PrE cells co-exist at non-synchronised stages of the cell cycle with associated fluctuation of pERK levels. We refer to the following reference https://www.ncbi.nlm.nih.gov/pubmed/31320324, cited in the context of Figure 2C, in which the ERK signalling pathway was investigated during early mouse development.

- If, as the authors propose, the primary effect of integrin inhibition is the downregulation of ERK signaling, then constitutively activating the pathway downstream of the integrin-receptor complex should rescue the PRE specification defect. For example an experiment treating integrin α5β1-inhibited embryos with a MEK activator could provide some insight for this causal link?

© 2026. Published by The Company of Biologists under the terms of the Creative Commons Attribution License (https://creativecommons.org/licenses/by/4.0/).

We thank the reviewer for the opportunity to clarify the methodological approach for this experiment. We consider activation of MEK via application of FGF4 should suffice, since we observe strong pERK protein, even in the presence of α5β1 inhibition. Furthermore, it is likely that using a MEK inhibitor will affect other systems within the cell, rather than clarifying this point. We have added the following few sentences to P6 to try to clarify this point: 'Our data suggest that localized ITGα5β1 activity is required in the ICM from E3.5 to E4.5 to achieve PRE cell lineage acquisition, thus implicating this essential receptor for fibronectin in regulating FGF/ERK activity and fate specification in PRE cells. While these results demonstrate that integrin signalling is required for ERK activation, other mechanisms, such as integrin-mediated clustering of FGF receptors or regulation of receptor trafficking, cannot be excluded.'

Figure 3
- In Figure 3A and within the text (method section), substrate stiffness should be stated instead of just soft or stiff. This is crucial for the reproducibility of the work and for understanding the specific mechanical window that supports NPRE cell derivation.

We thank the reviewer for pointing out this important omission. We have added values for the soft (3.5kPa) and stiff (160kPa) to figure 3A, highlighted in the text in the first paragraph of P7 and P16 in the methods section.

- In Figure 3D, the violin plot shows no statistically significant difference in colony area between fibronectin and fibronectin/laminin coatings, which appears to contradict the claim that fibronectin is superior based on the number and size of colonies. This discrepancy should be addressed; perhaps quantifying colony number in addition to area?

We apologise for this confusion. The reason behind the lack of statistically significant difference is the small number of colonies, particularly on laminin (3 for Laminin/Fibronectin, 14 for Fibronectin, t-test p value 0.1587). We have therefore added a graph quantifying the number of colonies in Figure 3, which also highlights the technical limitations associated with this experiment.

Figure 5
- In Figure 5C, it seems that there are more Nanog+ cells in P2 NPRE cells. Do these EPI-like cells arise from the de-differentiation of specified PRE precursors, demonstrating a remarkable degree of plasticity, or do they emerge from the selective expansion of a minor, undetected EPI-like population that persisted from the initial P0 culture? For example using a Sox17-CreERT2 driver to label PRE-like cells at an early passage and then tracking their fate after FGF removal would definitively distinguish between de-differentiation and selection of a pre-existing sub-population.

This is an interesting question, but we respectfully point out that we used Oct4, not Nanog, to label the cells in these experiments, which persists for longer than Nanog in maturing epiblast cells. The 2 Oct4 positive cells in question also express PRE markers, suggesting that they represent ICM cells that have not committed to EPI or PRE at this stage. Interestingly, de-differentiation was previously shown within mouse blastocyst chimeras during the segregation process, pretty much up until implantation (see Grabarek et al., 2012). We were unable to prevent expansion of EPI-like cells in the population. We appreciate the suggestion of labelling PRE-like cells at an early passage, but during the process of expansion we suspect they would progress to a more mature form of PRE, which would not be useful for our studies. We do appreciate the suggestion to create a Sox17-CreERT2 line, but again, during the expansion of such a line, the PRE cells will most probably have advanced developmentally beyond the stage of interest. We have rewritten this section of the discussion, highlighted in yellow on P11, to emphasise more strongly the limitations we and others have encountered during the process of trying to establish self-renewing, bona fide naïve PRE cell lines.

Figure 6
- The scatter plot in Figure 6E shows high variability in the number of contributing cells per embryo, ranging from a single cell to more than ten. The source of this variability is not discussed but could be linked to the inherent heterogeneity of the NPRE population?

© 2026. Published by The Company of Biologists under the terms of the Creative Commons Attribution License (https://creativecommons.org/licenses/by/4.0/).

As the reviewer suggests, we suspect that variability in differentiation status and contribution between the NPRE cells is indeed linked to the inherent heterogeneity of the population. The highlighted section on P11 is also relevant to this point; we thank the reviewer for pointing this out.

Clonality
The current derivation protocol starts from a population of 50-70 dissociated ICM cells, leading to a heterogeneous P0 population. Attempting to derive clonal NPRE lines from single cells would be highly informative. This would reveal whether the observed instability is an intrinsic property of all NPRE cells or if stable clones can be isolated from a heterogeneous starting pool.

We thank the reviewer for this suggestion and agree about the benefits of deriving clonal NPRE cell lines to improve the homogeneity of the population and select for stable clones. This experiment had indeed occurred to us as worthwhile and informative. Unfortunately, when we attempted clonal derivation, the individual cells did not survive or differentiated before they could be expanded. We attribute this to either the progressive tendency to differentiate that eventually restricted long term expansion for most of our lines or to unforeseen limitations in our derivation protocol which could be explored in future studies.

Some current blastoid models are relying on mESCs. Could the finding of this manuscript be applied to sorted PRE cells from blastoids to derive a more nascent population of PREs?

This is an excellent suggestion that could effectively be applied to blastoids made from naïve human pluripotent stem cells, which readily differentiate and organise into blastocyst-like structures, spontaneously generating and positioning trophectoderm and hypoblast cells, (see https://www.ncbi.nlm.nih.gov/pubmed/33957081). However, mouse blastoids require a mixture of trophectoderm stem cells and ESCs, and they subsequently struggle to produce more than a few PRE cells (see https://www.ncbi.nlm.nih.gov/pubmed/35803228, https://www.ncbi.nlm.nih.gov/pubmed/29720634). The small number of PRE cells present in mouse blastoids would therefore be a limiting factor for this suggested experiment.

Minor comments:

- the authors' claim of "reproducing the mechanical environment of the in vivo peri-implantation equivalent", may be an overstatement. The in vivo ICM is a highly complex, three-dimensional aggregate of cells with dynamic cell-cell and cell-ECM interactions. The authors' 2D hydrogel system, while a significant improvement over rigid plastic, is a simplification of this intricate niche. The language throughout the manuscript should be moderated to present the system as a step towards recapitulating the in vivo environment, rather than a faithful replica of it.

We thank the reviewer for pointing out the over-interpretation of our findings. We have moderated our claims throughout the text in the abstract, introduction and discussion, as suggested and swapped the word 'equivalent' for 'as closely as possible'.

- Figure 1B: Integrin gene expression from single ICM cells (EPI and PRE precursors) at E4.0. The number of embryos needs to be specified.

Thanks so much for pointing out this oversight. We have added the numbers of cells used for Figure 1B to the figure: 13 PRE cells and 14 EPI cells, and number of embryos: 3, in the figure legend. The origin of this data can be found in Yanagida et al., 2022 (https://pubmed.ncbi.nlm.nih.gov/35196500/)

Different PRE markers have been used even within one figure throughout the manuscript. Ex. Sup Fig 3C, Fig. 5D,E. Is that to diversify the markers? It would be better to include both rather than alternating between those.

In Figure 5D-E, SOX7 is used to track the development and progression of PRE cells since this is known to be a marker of a later stage. Our aim in Sup Fig 3C was to diversify the markers used, as suspected by the reviewer, as well as to ensure no overlap of host animals in which the antibodies were raised, requiring the combinations to be adapted.

© 2026. Published by The Company of Biologists under the terms of the Creative Commons Attribution License (https://creativecommons.org/licenses/by/4.0/).

- Consider including the expression of other ECM proteins, such as laminin, in Figure 1, particularly since it is further examined in Figure 3.

We thank the reviewer for this sensible suggestion. However, we had decided not to replicate the results from a recent paper from the Hiiragi lab (Kim et al. 2022, see reference list), which already described expression of several ECM proteins during the stages of interest in mouse embryos, but focussing more on cell positioning during early lineage segregation. Our decision not to duplicate that data also has the virtue of minimising animal usage.

- In section : Integrin activity affects lineage acquisition via FGF/ERK signalling, the data demonstrate a requirement for integrin signaling for proper ERK activation, but do not exclude other potential mechanisms, such as integrin-mediated clustering of FGF receptors or regulation of receptor trafficking. The text should reflect this ambiguity.

We apologise for this omission and have reflected the ambiguity in the text, mentioning the possibility of additional mechanisms, highlighted on P6, also quoted in the context of Figure 2 above.

- The abstract and introduction make claims about the potential for NPRE cells to integrate into blastoids. However, the experiments presented only show the formation of ICM-like aggregates with mESCs (Fig. 6A), not full blastoids that include a trophectoderm layer. The claims in the manuscript should be revised to more accurately reflect the data that is actually presented.

We apologise that our abstract and introduction are misleading; this was not our intention, so we thank the reviewer for pointing it out. We have rewritten both abstract and introduction to be more circumspect and more accurately to reflect the data.

Reviewer 2: SUMMARY OF THE ADVANCE MADE IN THIS PAPER AND ITS POTENTIAL SIGNIFICANCE TO THE FIELD

In recent years, several stem cell-based models of early mammalian development have been reported. Although such models possess considerable potential for advancing research within the scientific community, particularly in the investigation of human preimplantation and early postimplantation development, several key challenges must be addressed to facilitate their successful application. One of the most important is the assurance that the models can fatefully replicate the physiology and behaviour of their in vivo counterparts. This specific issue was addressed by Chalut and colleagues in their current manuscript. Namely, the authors aim was to derive cells in culture that are equivalent of pre-implantation primitive endoderm (PRE). PRE is one of the 3 key lineages that arise early in development and so far, the attempts to capture preimplantation PRE state were not fully successful.
To achieve their goal, Chalut and colleagues' approach was to re-create in the cell culture the condition equivalent to those found in the ICM of the developing embryo. By testing various soft and stiff substrates in combination with most suitable extracellular matrix elements the authors were able to find the mechano-environment that supports derivation of PRE equivalent population.

We thank the reviewer for this positive and accurate summary of our work, and for understanding the challenges to derive *in vitro* cell lines from early embryos.

SUGGESTIONS TO AUTHORS

The protocols and new information about the combined effects of integrin and mechano-environment on PRE and Epi maintenance included in this manuscript can be potentially very useful for the scientific community working on cell-based models of early mammalian development. However, several issues need to be addressed to help the reader to take full advantages of the data provided here.

© 2026. Published by The Company of Biologists under the terms of the Creative Commons Attribution License (https://creativecommons.org/licenses/by/4.0/).

We appreciate the reviewer's comment on the potential of our work and thank them for the suggestions to improve its clarity. We have performed additional measurements and added information in various figures following their advice.

Major issues

1. There is no clear explanation why the authors chose to specifically look at integrins but no other elements of ECM?

We are grateful to the reviewer for pointing this out. We chose integrins because they are well known as cell surface receptors mediating cell-ECM interaction and adhesion. The most obvious other ECM and receptor pairing we would see at that stage is HA-CD44, however, neither the synthesis gene (HAS1-3), nor receptor gene (CD44) are expressed according to the E3.75 dataset from Yanagida et al., 2022 (see reference list). Our first objective was to uncover the ECM environment that would best support the PRE, hence starting by characterising integrins expression to see what type of ECM these cells are primed to bind. Each integrin heterodimer, consisting of an alpha and beta subunit, has a high specificity for particular ECM ligand(s), (e.g. ITGb1α6 binds Laminin, ITGb1α5 binds Fibronectin – https://www.mdpi.com/1422-0067/19/2/449), expression data hence giving a good idea what ECM ligand is native for their environment. Based on the integrin expression, PRE cells expressed fibronectin-binding integrins, whereas EPI cells expressed laminin-binding integrins, suggesting derivation of each lineage could be improved by distinctive ECM coatings. This was supported by our experimental data (Figure 3). We have added a summary of this explanation into the text:

'We therefore characterised ICM integrin expression to choose the most suitable extracellular matrix (ECM), and modified substrate stiffness to mimic the *in vivo* condition'

2. In the first section of the Results (Integrin α5β1 activity is necessary for PRE cell fate specification), the authors wrote: 'At E4.5, the difference in intensity of ITGα5 became more evident, and active ITGβ1 was clearly localized to the boundary between EPI and PRE' - can the authors provide some kind of measurements (plus nr of embryos/cells analysed) to support their claim?

For clarity, we have now inserted the number of embryos analysed for each figure in the corresponding image panel in the revised manuscript. In addition, we have measured the intensity of ITGα5 signal across the PRE and across the EPI through image analysis in FIJI, following the example from Kim et al, 2022 (https://journals.biologists.com/dev/article/149/1/dev200140/273994/ECM-integrin-signalling-instructs-cellular). In Figure 1C, there are now two graphs comparing the intensity of ITGα5 signal between the EPI and PRE cells, in both the embryo and ICM aggregate chosen for this figure. We have repeated the measurement in additional embryos and ICMs (see graphs below). However, due to the difference in cell sizes across samples, we decided to show only one example in the main figure.

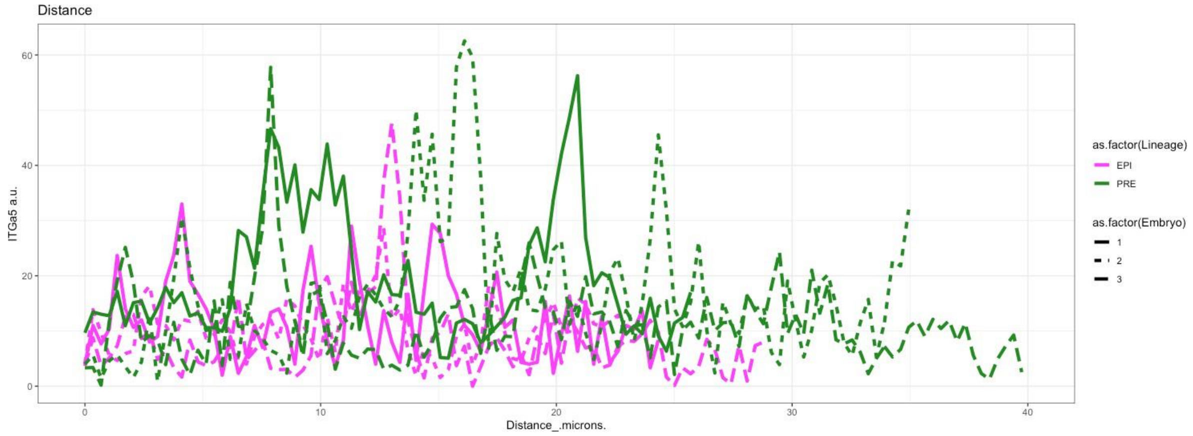

Revisions Figure 1. Comparison of ITGα5 signal between EPI and PRE cells in E4.5 embryos.

© 2026. Published by The Company of Biologists under the terms of the Creative Commons Attribution License (https://creativecommons.org/licenses/by/4.0/).

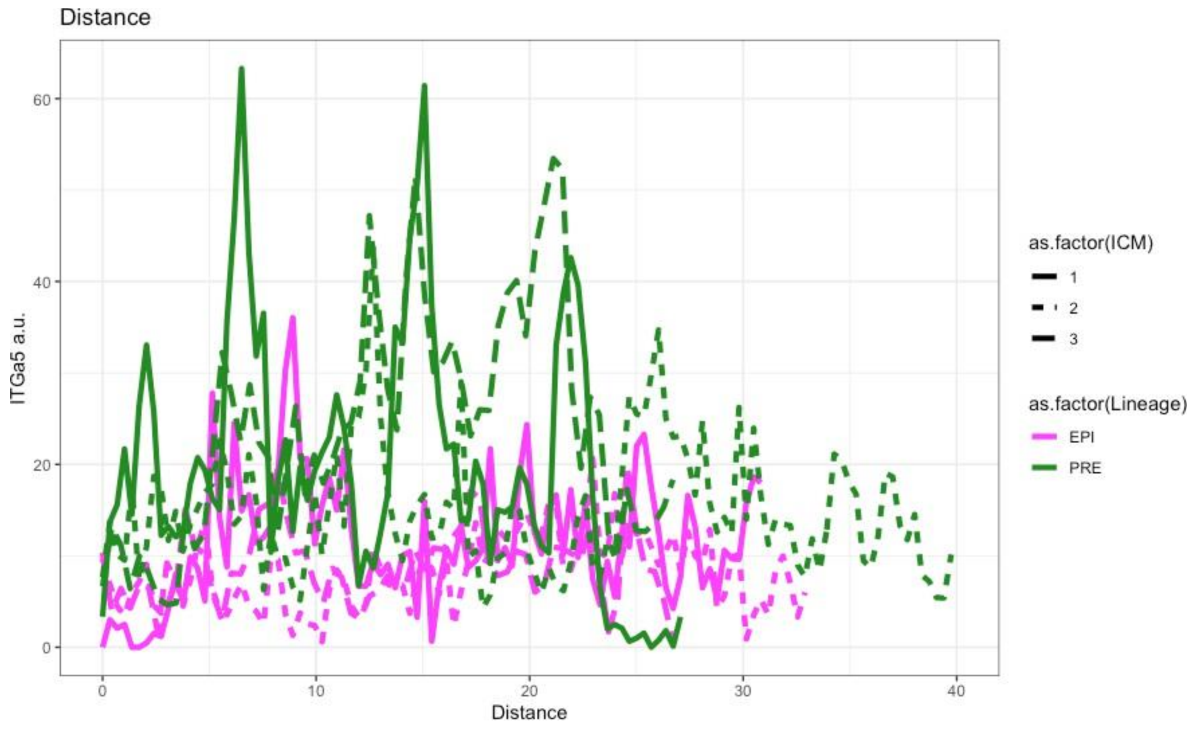

Revisions Figure 2. Comparison of ITGα5 signal between EPI and PRE cells in E4.5 ICM aggregates.

3. Similarly, in the same section of the Results, the sentence 'However, as with the expression pattern of ITGα5, fibronectin expression was clearly localized in the sorted PRE beside the blastocoel in E4.5 embryos and outer cells of E4.5 ICM aggregates' also needs to be supported by measurements (plus nr of embryos/cells analysed).

In response to this helpful suggestion, we have now inserted the number of embryos analysed within each relevant panel in the revised manuscript. We also measured the intensity of fibronectin signal starting from the polar TE towards the blastocoele (EPI -> PRE). This data is now presented in the revised Figure S1D.

4. The measurements should also be provided for the data currently presented in Fig 1E. (sentence 'Clear localisation of fibronectin in PRE cells at E4.5 was lost when combined with ITGα5β1 inhibition, remaining ubiquitously expressed in the ICM (Fig. 1E)'.

We have provided the number of embryos analysed in the revised manuscript. We also measured the intensity of the fibronectin signal by drawing a line in the embryos from the polar TE towards the blastocoele, across the ICM from EPI to PRE cells and include the measurement from the images in Figure 1E in the revised manuscript. We also analysed additional control (4) and treated (4) embryos, and the measurements can be seen below. We decided to show only one embryo for each in the manuscript for clarity.

© 2026. Published by The Company of Biologists under the terms of the Creative Commons Attribution License (https://creativecommons.org/licenses/by/4.0/).

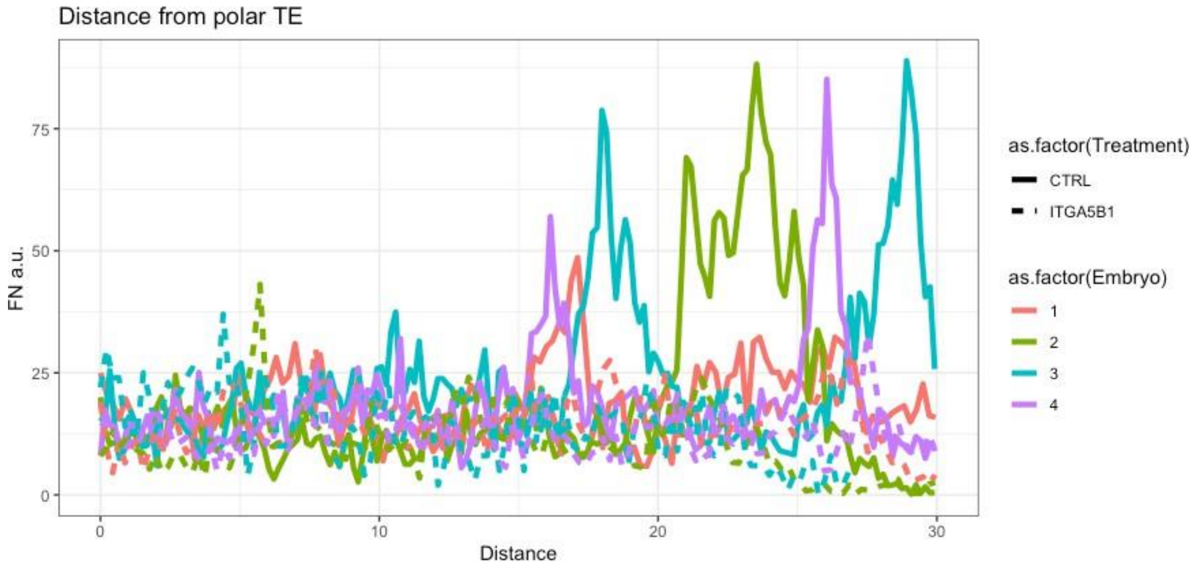

Revisions Figure 3. Comparison of fibronectin (FN) signal across the ICM, from the polar TE towards the blastocoele in both control and treated embryos.

5. Conclusion from figure 1 and 2 is basically the same in the main text. Could the authors re-write this part to highlight what exactly is the main message from the data include in fig 2?

We apologise that our original explanation was unclear. In Figure 1 we analyse integrin expression, fibronectin, and the potential role of integrin in cell fate acquisition and maintenance. In Figure 2, we study the role of FGF signalling as the main signalling pathway involved in PRE differentiation. In response to both reviewers, we have added the following text to clarify the distinction between the motivation and investigation presented in Figures 1 and 2: ´While these results demonstrate integrin signalling is required for ERK activation, other mechanisms such as integrin-mediated clustering of FGF receptors or regulation of receptor trafficking cannot be excluded' highlighted in yellow on P6, to emphasise that this section focuses on ERK signalling and integrin activity, and also 'Our data suggest that localized ITGα5β1 activity is required in the ICM from E3.5 to E4.5 to achieve PRE cell lineage acquisition, thus implicating this essential receptor for fibronectin in regulating FGF/ERK activity and fate specification in PRE cells', also highlighted in yellow on P6. We thank the reviewer for pointing out the lack of clarity of this section in our initial submission and for the opportunity to improve the manuscript.

6. What was the timing in experiments presented in fig S2?

We apologise for the omission of this detail. The length of the experiment was 30-32 hours, from E3.25 to E4.5. This information has now been added to Figure S2.

7. What was the reason for 'a significant decrease in the number of PRE cells in embryos subjected to integrin inhibition' (data presented in Fig. 2A-B)? Was it due to failure of specification or for example due to poor survival of PRE cells in such conditions?

Embryos in Figure 2A do not show an increase in cell death which would suggest a problem with PRE specification, however, poor survival under this treatment could add to the decrease in PRE cells compared to FGF only treatment. We also show a reduction in PRE cells under integrin inhibition in Figure 1. In this case, we did not see dead cells, but rather cells not showing the appropriate markers for EPI or PRE. The total ICM number, measured in Figure S1D, does not change between control and integrin inhibition which prompt us to hypothesise the issue is failure in PRE specification rather than survival.

8. The presentation of Fig 2B not easy to understand. What do the different boxes in y-axis represent? This should be explained better in the figure legend.

© 2026. Published by The Company of Biologists under the terms of the Creative Commons Attribution License (https://creativecommons.org/licenses/by/4.0/).

We thank the reviewer for pointing out this lack of clarity and have therefore deleted the boxes on the right in the figure, since they unnecessarily duplicate information on the x axis. We apologise for this misunderstanding.

9. In the part of the Results 'Soft FN-coated substrates promote PRE growth' the authors mentioned that Lif removal resulted in poor survival and cited fig S3A but this figure does not have data on Lif removal.

We apologise for this careless mistake and have changed the wording to 'data not shown'. This is because the cells did not survive in the absence of LIF.

10. In the part of the Results 'Soft FN-coated substrates promote PRE growth' the authors stated: 'Under FGF4 treatment, ICM outgrowths on soft hydrogels were enriched with PRE, while those on TCP also contained a TE population (Fig. S3C).' How was the enrichment calculated (fig S3C only contains representative images)?

We realise that this section is a bit confusing. This is because there are no TE cells on soft substrates. Our hypothesis therefore focuses on the fact that the equivalent of the cells becoming TE on stiff substrates take the alternative route of become PRE cells on the soft. We have adjusted the wording in the text: 'On TCP, ICMs attached and formed outgrowths of the three pre-implantation fates: TE, EPI and PRE, while on soft hydrogels, there was no detectable TE (Fig. S3C). Under FGF4 treatment, ICM outgrowths on soft hydrogels produced predominantly PRE cells, while those on TCP also contained a TE population (Fig. S3C).' highlighted in yellow on P7. Our conclusion was drawn based on the lack versus presence of TE markers in the representative images.

11. From fig 5 B - it is not that clear to me which passage represent which stage of in vivo development. Could the authors provide a common timescale (with embryonic days (E) clearly marked on the timeline) for in vivo and in vitro (passages) and add it to the figure? Matching the exact passage to the desirable developmental stage will be important if the NPRE cells were to be used to create more sophisticated cell-based models of mammalian pre and peri-implantation development. It would also be good to discuss in the main text why while the progression of developmental stages is preserved in the subsequent passages, the length of the time for each in vivo and in vitro stage varies.

We apologise for the complexity of this figure and have now amended the diagram to clarify the equivalence between *in vivo* and *in vitro* counterparts. Once the cells have reached a later passage and no longer have plasticity, they do not continue to develop any further. The unstable populations are those with greater plasticity at early stages of development and at earlier passages.

12. The way the figure description is written/organised in the current version of the manuscript is often very confusing. For consistency, all the figures legend should be structured in the same way for the same type of experiments/treatments. Also, all necessary information needs to be included.

We thank the reviewer for their comments and have worked on the figure descriptions to improve clarity. We have added Figure 1 legend as an example below. This figure legend now contains information about the number of cells and embryos analysed and uses the same wording to describe all the 'Representative confocal images' present in the figure. 'Nucleus is shown with Hoechst' replaces the other versions used to describe the same nuclear staining.

© 2026. Published by The Company of Biologists under the terms of the Creative Commons Attribution License (https://creativecommons.org/licenses/by/4.0/).

Figure 1

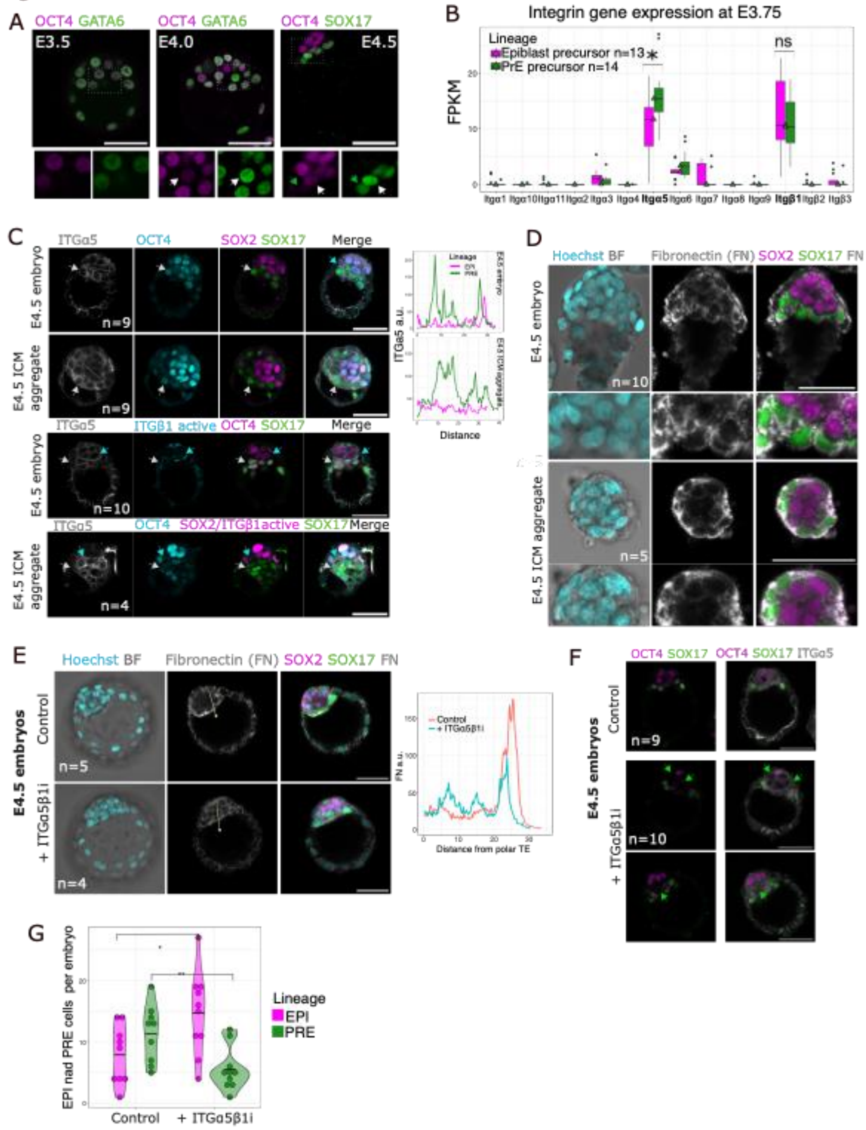

Figure 1. Differential integrin expression and activity between EPI and PRE during blastocyst development. A. Representative confocal images of pre-implantation mouse embryos from E3.5 to E4.5 immunostained for OCT4 (magenta, EPI) and GATA6/SOX17 (green, PRE). B. Integrin gene expression from single ICM cells (14 EPI and 13 PRE precursors) at E3.75 from 3 embryos. Statistical significance was assessed using a t-test (* $p < 0.05$, ns $p > 0.05$). C. Representative confocal images of E4.5 mouse embryos or E4.5 ICM aggregates – made of 3 E3.5 ICMs – immunostained for ITGα5 (white), active ITGβ1 (cyan or magenta), OCT4 (cyan or magenta), SOX2 (magenta) and SOX17 (green). White arrows indicate higher ITGα5 expression, cyan arrows indicate localized active ITGβ1 expression. Representative intensity profile of ITGα5 across the EPI (marked as a magenta line on the confocal image on the left) and across the PRE (marked as a green line on the confocal image on the left). D. Representative confocal image of E4.5 mouse embryos and ICM aggregates immunostained for Fibronectin (FN) (grey), SOX2 (magenta) and SOX17 (green). Nucleus is shown with Hoechst. E. Representative confocal images of E4.5 mouse embryos cultured in the absence or presence of antibodies to block ITGα5β1 activity (CD29 and CD49e) and immunostained for SOX2 (magenta), SOX17 (green) and FN (grey). Representative intensity profile of fibronectin (FN) in control (cyan) and treated (red) embryos from the polar TE across the EPI into the PRE and the blastocoele (marked as lines on the confocal images on the left, arrowhead indicates the end of measurement). F. Representative confocal images of E4.5 mouse embryos cultured in the absence or presence of antibodies to block ITGα5β1 activity (CD29 and CD49e) and immunostained for OCT4 (magenta), SOX17 (green), ITGα5 (grey)

© 2026. Published by The Company of Biologists under the terms of the Creative Commons Attribution License (https://creativecommons.org/licenses/by/4.0/).

and active ITGß1 (white). Nucleus is shown with Hoechst. Green arrows point towards central ICM cells expressing neither OCT4 nor SOX17. Scale bar is 50µm in all images. G. Violin plot showing the average total cell number of each lineage in control and treated embryos. The numbers of embryos used for each experiment are written within the relevant panels. Statistical significance was assessed using a t-test (* $p < 0.05$, ** $p < 0.005$).

* For example, panel F in Suppl fig 5 - the figure legend is not consistent with the panel D and E descriptions. Namely, it does not include information about the surface type and passage nr as provided in panels D and E. Why?

We apologise for the missing information in Sup Figure 5F which has now been added: P8 for NPRE FGF+ and P25 for NPRE FGF-.

'F. Representative confocal image of P8 FGF+NPRE cells (top panel) and P25 FGF-NPRE cells (bottom panel) immunostained for SOX17 (green), OCT4 (magenta), active ITGß1 (cyan) and ITGα5 (grey) cultured on FN-coated soft hydrogels.'

* Fig 6A - in the figure legend PRE cells are mentioned (which may indicate PRE cells isolated from ICM), whereas in the figure itself they are named NPRE - which suggests that they are the culture derived cells.

We apologise; the error in Figure 6A is a typo, it should say NPRE cells in the legend, which we have now corrected.

'A. Representative confocal images of ICM aggregates made of 30 NPRE cells and 20 mESCs 8, 24 and 48 hours after initial seeding. Pdgfrα $^{H2B\text{-}GFP/+}$ FGF+NPRE cells (green) and mESCs immunostained for OCT4 (magenta).'

* Fig S6C description is very vague

We appreciate the description is very short. We have added more information about the PDGFRα-GFP signal and an indication that these cells will be the ones used for injection in Figure 6. More information about the method can be found in the MM.

'C. NPRE Pdgfrα $^{H2B\text{-}GFP/+}$ cells transfected with mKO (magenta) for injection in Figure 6C.'

* How many embryos were injected with NPRE cells in experiments described in FigS6F?

We apologise for the lack of information in the manuscript regarding this experiment and thank the reviewer for noticing this omission. We have now added the following information into Figure S6F: '17 morulas were injected with NPRE FGF+ P15, and 9 morulas were injected with NPRE FGF+ P6.'

13. In the part titled "NPRE FGF+ cells can contribute to host blastocyst PRE and aggregate with mESCs" the authors wrote: "(…) we separated the cells based on morphology and cultured them as two distinct cell lines for one passage prior to aggregation" - more details is needed (like what were the morphological criteria, were the cultures homogenous after separation, how this homogeneity was assessed etc.)

Thanks for the opportunity to clarify how we categorised the cells. The morphology differences can be observed in Figure S5B. EPI-like cells form ESC-like colonies with bright, round edges while NPRE have rough edges. We also checked the fluorescence and PDGFRα-GFP signal of NPRE cells. Cultures were homogeneous after separation for at least three passages before freezing.

14. In the Aggregate formation part of MM it is described that NPRE FGF+ cells were mixed with ESCs in different proportions than NPRE FGF-. Can the authors provide the rationale why?

We thank the reviewer for the careful reading of the MM section; however, we do not see what is suggested by their comment. The text read 'ICM-like aggregates were formed by mixing 20 NPRE FGF+ cells with 15 mESCs, or by mixing 15 EPI-like NPRE FGF- cells with 20 PRE-like NPRE

© 2026. Published by The Company of Biologists under the terms of the Creative Commons Attribution License (https://creativecommons.org/licenses/by/4.0/).

FGF-cells'. Perhaps the order of the lineages has led to confusion? Therefore, we have changed the order to present the PRE-like component of the aggregate first, and the EPI (mESCs, EPI-like NPRE FGF- cells) last in both instances.

Minor points

1. The reasons for adding ICM aggregate data to fig 1 becomes apparent only after reading the whole manuscript. The authors may want to consider whether fig 1 is the best place for those data.

ICM aggregates are used to characterise the integrin expression in early embryos and therefore need to appear early in the manuscript. We have rewritten the manuscript to explain why they are necessary earlier on: 'We therefore characterised ICM integrin expression to choose the most suitable extracellular matrix (ECM), and modified substrate stiffness to mimic the *in vivo* condition.' Highlighted in yellow on P4.

2. Fig 1G can be moved to supplementary figures as it does not necessarily add more info than fig 1H

We thank the reviewer for this suggestion and have now moved 1G to the supplementary material.

Second decision letter

MS ID#: dev.205226R1

MS TITLE: Harnessing the murine inner cell mass mechanical environment enhances derivation of in vitro nascent primitive endoderm precursor cells

AUTHORS: Elena Corujo-Simon; Siiri Salomaa; Lawrence E. Bates; Ayaka Yanagida; William Mansfield; Hannes Becher; Jennifer Nichols; Kevin J. Chalut

Dear Dr Nichols,

I have now received all the referees reports on the above manuscript, and have reached a decision. The referees' comments are appended below.

The overall evaluation is positive and we would like to publish a revised manuscript in Development, provided that the referees' comments can be satisfactorily addressed. Please attend to all of the reviewers' comments in your revised manuscript and detail them in your point-by-point response. If you do not agree with any of their criticisms or suggestions explain clearly why this is so. If it would be helpful, you are welcome to contact us to discuss your revision in greater detail. Please send us a point-by-point response indicating your plans for addressing the referees' comments, and we will look over this and provide further guidance.

Reviewer 2

SUMMARY OF THE ADVANCE MADE IN THIS PAPER AND ITS POTENTIAL SIGNIFICANCE TO THE FIELD

In the revised manuscript, the authors have adequately addressed all the questions posed by this reviewer, and it is my pleasure to recommend this work for publication. I have only a few additional minor points:

Please provide information on how many biological and how many technical replicates contributed to the data presented in Figures 3, 4, 5, and Supplementary Figures 3 and 5.

© 2026. Published by The Company of Biologists under the terms of the Creative Commons Attribution License (https://creativecommons.org/licenses/by/4.0/).

Please ensure that the passage number is reported for all experiments using NPRE cells. It is included for most figures, but there are some omissions.

I am unable to find information regarding the nuclear staining procedure for embryos and cells in the Materials and Methods section.

Second revision

Author response to reviewers' comments

Reviewers' comments:

- Please provide information on how many biological and how many technical replicates contributed to the data presented in Figures 3, 4, 5, and Supplementary Figures 3 and 5.

We thank the reviewer for pointing out this omission.

We have added the following information to Figure 3: A) FN n=6, Laminin n=3, FN/Laminin n=2; Stiff hydrogels n=1, TCP n=3 and inserted the following information into the Figure legend: A) The number of biological repeats is specified in the right side of the figure, the derivation was performed on soft hydrogels a total of 14 times (9 on FN, 2 on FN/Laminin and 3 on Laminin); on stiff hydrogels 2 times, and on TCP 3 times.

To Figure 4 we have added A minimum of 5 colonies were imaged per passage per ECM

To Figure 5A, under NPRE FGF- we state the number of derivations as n=4, and for NPRE FGF+ n=6. To the end of the legend, we have added A minimum of 5 colonies were imaged per passage per condition.

To Supplementary Figure 3, we have added number of ICMs seeded: n=3 under the images for B and n=3 under the images for C.

To Supplementary Figure 5 legend, we have added A minimum of 5 colonies were imaged per passage per ECM to the end of the legend, and also to the legend in D) Derivation on FN TCP for FGF- cells was performed twice, and for FGF+ 3 times.

- Please ensure that the passage number is reported for all experiments using NPRE cells. It is included for most figures, but there are some omissions.

Apologies for this oversight. We have added the following information:

Figure 4A: P0 is indicated in the figure legend, but for clarity we have also inserted P0 to the figure itself.

For Figure S6C we have inserted 'at P4 were used' and for Figure S6D 'at P3'

- I am unable to find information regarding the nuclear staining procedure for embryos and cells in the Materials and Methods section.

We apologise for this oversight, and have now inserted on page 12 of the manuscript in the M+M section:
"Secondary antibody incubation was performed together with Hoechst (1:2000) for nuclear staining for 1-2 hour at RT in the dark…"

© 2026. Published by The Company of Biologists under the terms of the Creative Commons Attribution License (https://creativecommons.org/licenses/by/4.0/).

For page 12 of the manuscript, for immunostaining of cells, we inserted “The next day, cells were washed 3 times in PBST for 15 min prior to secondary antibody and Hoechst incubation for 1-2 hours.”

Third decision letter

MS ID#: dev.205226R2

MS TITLE: Harnessing the murine inner cell mass mechanical environment enhances derivation of in vitro nascent primitive endoderm precursor cells

AUTHORS: Elena Corujo-Simon; Siiri Salomaa; Lawrence E. Bates; Ayaka Yanagida; William Mansfield; Hannes Becher; Jennifer Nichols; Kevin J. Chalut

Dear Dr Nichols,

I am happy to tell you that your manuscript has been accepted for publication in Development, pending our standard publication integrity checks.

© 2026. Published by The Company of Biologists under the terms of the Creative Commons Attribution License (https://creativecommons.org/licenses/by/4.0/).
